# MediSim: Multi-granular simulation for enriching longitudinal, multi-modal electronic health records

**DOI:** 10.1016/j.patter.2025.101261

**Published:** 2025-05-08

**Authors:** Brandon Theodorou, Cao Xiao, Lucas Glass, Jimeng Sun

**Affiliations:** 1 Department of Computer Science, University of Illinois at Urbana-Champaign, Urbana, IL, USA; 2 GE Healthcare, Seattle, WA, USA; 3 IQVIA, Durham, NC, USA

**Keywords:** machine learning in healthcare, generative modeling, imputation, augmentation, multi-modal EHR simulation, autoregressive generative modeling, data imputation and augmentation, reinforcement learning for synthetic data, clinical notes and imaging synthesis

## Abstract

We introduce MediSim, a multi-modal generative model for simulating and augmenting electronic health records across multiple modalities, including structured codes, clinical notes, and medical imaging. MediSim employs a multi-granular, autoregressive architecture to simulate missing modalities and visits and iterative, reinforcement learning-based training to improve simulation in low-data settings. Additionally, it utilizes encoder-decoder model pairs to handle complex modalities like notes and images. Experiments on outpatient claims and inpatient ICU datasets have demonstrated MediSim’s superiority over baselines in predicting missing codes, creating enriched data, and improving downstream predictive modeling. Specifically, MediSim improved over 74% on missing code prediction, enabled up to 65% better downstream predictive performance compared to original deficient records missing either some visits or entire data modalities, and successfully produced realistic note and X-ray samples for use in downstream tasks. MediSim’s ability to generate comprehensive, high-dimensional EHR data has the potential to significantly improve AI applications throughout healthcare.

## Highlights

• MediSim enriches EHRs by simulating visits, modalities, and complex data types
• Multi-granular architecture enhances both temporal and modality-level predictions
• Reinforcement learning boosts simulation quality in low-data clinical settings
• MediSim improves downstream model F1 scores by up to 65% over raw EHR data

## The bigger picture

Electronic health records (EHRs) hold immense potential for transforming clinical care and medical research through data-driven approaches. However, their usefulness is often hampered by incomplete, inconsistent, or siloed data. Patients routinely receive care across multiple institutions, leading to fragmented records with missing visits, absent clinical notes, or unavailable imaging data. These data gaps present significant challenges for developing and deploying robust machine learning models in health care, especially when high-quality training data are limited.

MediSim addresses this widespread issue by offering a multi-granular generative framework capable of enriching deficient EHRs. Rather than relying on conventional imputation or synthetic data generation techniques that often fall short in realism or versatility, MediSim uses a deep autoregressive model with reinforcement learning-based training to simulate missing information across both time and modality. By generating realistic and diverse records that span structured medical codes, clinical notes, and medical images, MediSim enables the creation of comprehensive patient profiles. This can bolster the performance of downstream predictive models, improve data equity across institutions, and support privacy-preserving analytics. As the healthcare industry continues to expand the use of AI, solutions like MediSim play a pivotal role in making imperfect real-world data more usable, reliable, and inclusive.

## Teaser

Theodorou et al. introduce MediSim, a multi-modal generative framework for enriching electronic health records (EHRs) by simulating missing visits, modalities, and complex data such as notes and images. Leveraging autoregressive modeling and reinforcement learning, MediSim significantly improves predictive performance on downstream tasks and offers a practical solution to common EHR deficiencies, making it a powerful tool for robust AI development in healthcare.

## Introduction

Electronic health records (EHRs) have been adopted in the United States by more than 90% of hospitals for inpatient and outpatient medical care.^1^ They provide the foundation for advancing the development of machine learning and digital health solutions that improve the quality and efficiency of care. EHRs consist of longitudinal sequences of clinical events, including high-dimensional medical codes and unstructured clinical note text that record the diagnoses, medications, and procedures patients receive during each hospital visit. Machine learning models have been developed on EHR data for disease risk prediction,^2^^,^^3^^,^^4^^,^^5^ health monitoring,^6^^,^^7^ disease phenotype learning,^8^ and treatment recommendation.^9^^,^^10^ These models operate on the assumption that EHR data provide a comprehensive representation of patients’ health conditions.

It is widely acknowledged that there are data-quality issues with many existing EHR datasets.^11^^,^^12^^,^^13^ They often exhibit temporal missingness (e.g., short record length) or missing data modalities (e.g., clinical notes or lab results are missing due to data access constraints) or both,^14^ and high-quality data are often limited to only a small subset of patients.

To address this data-quality issue, especially with respect to impeding the utility of such data to train effective machine learning models for downstream tasks, we propose the task of generative EHR enhancement, in which we simulate missing (or even just absent) fields using predictive or generative methods to extend records and improve their capabilities for supporting downstream use.

Some approaches have attempted to rectify these data-quality issues through generative models for direct EHR generation, like the generative adversarial network (GAN)-based method of Zhang et al.^15^ or the variational autoencoder (VAE)-based model of Biswal et al.^16^ However, these models tend to produce unrealistic data compared to original records, struggle when dealing with small training datasets, and lack the ability to enhance or repair real data. Apart from GAN- and VAE-based models, all predictive modeling techniques can theoretically be adjusted to fill in missing medical codes over time,^2^^,^^3^^,^^4^^,^^5^ and this simulation can even improve downstream performance in some cases. However, in practice, these models can only handle a limited format of missing data types, and their performance often significantly decreases, especially when there are limited training data available.

To fill the technical gap, we propose MediSim, a multi-granular deep autoregressive data generation architecture wrapped in an iterative, reinforcement learning-based training regime. MediSim is enabled by the following technical contributions.

• Autoregressive model to extend sequences and fill in missing code modalities: The architecture of the autoregressive model leverages a pair of multi-granular autoregressive modules designed to exploit both visit- and code-level structures within a patient record. These modules allow code-level generation to be conditioned on past visits and medical codes within the same time step, enabling flexible, bi-directional simulation of both missing visits and missing codes within present visits.
• Iterative, reinforcement learning-based training for limited data: MediSim incorporates a special training procedure that draws inspiration from reinforcement learning from human feedback, using a discriminator-style reward model trained to distinguish real from synthetic data. This approach further promotes the high-quality generation of realistic data and effectively addresses the challenge of limited training data volume.
• Paired models for encoding and generating additional modalities: The core architecture of MediSim embeds structured medical history to simulate missing and future medical codes and visits. Encoder-decoder model pairs are employed to handle additional modalities, such as clinical notes and medical images.

We evaluated MediSim in two different types of settings: longitudinal structured EHR data and complex modalities such as clinical notes and chest X-ray images.

For the longitudinal structured EHR evaluation, MediSim consistently outperformed baselines. Specifically, MediSim achieved up to a 3.9× improvement in longitudinal code prediction F1 score (from 0.095 to 0.479) over baseline predictive models. Additionally, we achieved a 74% enhancement (from 0.333 to 0.582) in same-visit modality code prediction F1 scores over baseline imputation models, showing an ability to predict missing medical codes more accurately. We then show the value of the data enrichment task itself, demonstrating that modality-augmented data aided downstream models in achieving an average 7% performance increase (from 0.244 to 0.262 in F1 score) over original real data, while extended data enabled a 65% average performance boost (from 0.316 to 0.522 in F1 score) over real data.

For the unstructured modality evaluation, we showed that MediSim is able to effectively handle additional, more complex modalities such as clinical note^17^ and chest X-ray^18^ datasets. We show that MediSim can leverage this multi-modal data to improve structured data simulation (reducing perplexity by up to 5%) and also effectively impute missing modalities by generating realistic samples.

## Results

### Problem formulation

We briefly introduce the terminology and notations of EHR data and introduce our task of EHR simulation and enhancement.

#### EHR data

We represent patient record R as a sequence of visits over time as given by Equation 1,

(Equation 1) $$R=V^{\left(1\right)},V^{\left(2\right)},\ldots ,V^{\left(T\right)}\text{,}$$

where each visit $V^{\left(t\right)}$ is then composed of a varying number of medical codes and other medical modalities such as clinical notes, imaging scans, and additional information. The medical codes are split into diagnoses D, procedures P, and medications M. The additional modalities may include a note $n^{\left(t\right)}$ containing unstructured text describing the contents of the codes, as well as other details regarding the visit. It may also include an image $i^{\left(t\right)}$ that visually represents the condition and findings of organs, tissues, and other anatomical structures. Finally, it may encompass any more complex medical modality that cannot be represented as a set of codes or variables. Continuous variables such as lab values and time spans could also be included either as complex modalities or as structured medical codes through discretization or other representation processes, although we omit such discussion here. To sum up, we represent a visit as the set of diagnosis codes $d_{i}^{\left(t\right)}$, procedure codes $p_{i}^{\left(t\right)}$, medication codes $m_{i}^{\left(t\right)}$, note text $n^{\left(t\right)}$, imaging scans $i^{\left(t\right)}$, and any other medical modalities at that time as given by

$$V^{\left(t\right)}=\left(d_{1}^{\left(t\right)}\cdots d_{\left|V_{D}^{\left(t\right)}\right|}^{\left(t\right)},p_{1}^{\left(t\right)}\cdots p_{\left|V_{P}^{\left(t\right)}\right|}^{\left(t\right)},m_{1}^{\left(t\right)}\cdots m_{\left|V_{M}^{\left(t\right)}\right|}^{\left(t\right)},n^{\left(t\right)},i^{\left(t\right)},\cdots \right)\text{.}$$

#### EHR deficiencies

A patient record and its corresponding representation, denoted by R, may have different types of deficiencies.A longitudinally deficient record $R_{l}$ (here, l stands for longitudinal) means that some visits are missing and only a few visits are present in $R_{l}=\left[V^{\left(1\right)},V^{\left(2\right)},\ldots ,V^{\left(T^{\prime }\right)}\right]$, where $T^{\prime }<T$. For example, a patient may switch to a different clinic without direct access to prior encounters.

A modality-deficient record ${\;R}_{m}$ (here, m stands for modality) means that some modalities, such as medications or medical imaging data, may be missing. This kind of missing modality occurs quite often due to the inaccessibility of certain data modalities or the data being in another provider’s environment. For example, diagnosis information might be present, but procedures and medications can be incomplete or entirely missing from the datasets. In this case, $R_{m}$ will have full-length T, but each visit $V^{\left(t\right)}$ will be missing codes corresponding to procedures and medications along with other unstructured modalities.

These two deficiencies can also co-occur as a dually deficient record $R_{l/m}$ (here, l/m stands for longitudinal and modality) for a record that is missing both visits and modalities.

**Figure 1:**
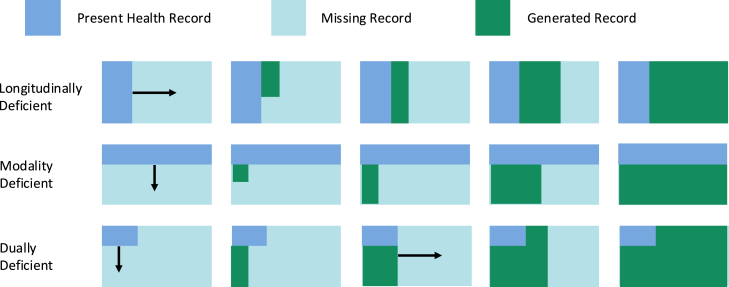
A visualization of the simulation and data extension process The process of extending the three types of EHR data deficiencies involves the following methods. Longitudinally deficient EHRs (top) are conditioned on the actual visits, and new visits are generated from there. Modality-deficient EHRs (center) generate visit history conditions before using the code-level architecture to complete the existing visits and then continue generating subsequent visits with missing modalities. Finally, EHRs with both types of deficiencies (bottom) complete the modalities of the existing visits before generating entirely new visits from there.

The three classifications of deficient records can be seen in the first column of Figure 1 for a visual reference.

#### Deficient EHR enhancement task

Our task is to generate a complete record $R^{\prime }$, which is a simulated patient of full length and modalities that looks like R and offers better utility than the original deficient $R^{*}\in \left\{R_{l},R_{m},R_{l/m}\right\}$. To accomplish this, our MediSim model learns and samples from the probability distribution $P\left(R|R^{*}\right)$, which represents all possible records given all possible deficient records. We provide a table of notations and their definitions for easy reference in Table S1.

### Experimental design

We evaluate MediSim and compare it to a number of baselines in a series of experiments on two EHR datasets. We design experiments to answer the following questions.

Q1: Is MediSim effective at accurately predicting codes to extend the length and impute missing medical code modalities of EHR sequences?
Q2: Can MediSim’s enriched data benefit downstream disease classification and prediction tasks?
Q3: Does MediSim effectively utilize and generate complex medical modalities?

We provide some brief pertinent experimental information here, and we provide more complete details in Section.

#### Data

We use two datasets for training MediSim and other baseline models in our experiments. The first is IQVIA’s proprietary outpatient EHR dataset consisting of comprehensive real-world US claims data. The second is an inpatient EHR dataset from the public Medical Information Mart for Intensive Care III (MIMIC-III) intensive care unit ICU stay data.^17^ Both datasets share a similar patient data format as a series of visits, and both contain each of the three modalities of medical codes in diagnoses, procedures, and medications. However, the outpatient EHR data are significantly larger and have more visits per patient, while the inpatient EHR data contain significantly more medical codes in the procedure and medication categories along with additional, more complex modalities such as imaging and text.

#### Baseline comparisons

We break our baseline models down into two groups:

(1) The temporal extension baselines include GPT,^19^ LSTM,^20^ SynTEG,^15^ RETAIN,^3^ CONAN,^4^ and Dipole.^5^
(2) The modality imputation baselines are logistic regression (LR), neural network (NN), and cascaded residual autoencoder (CRA).^21^

### Q1: Accurate EHR simulation

**Figure 2:**
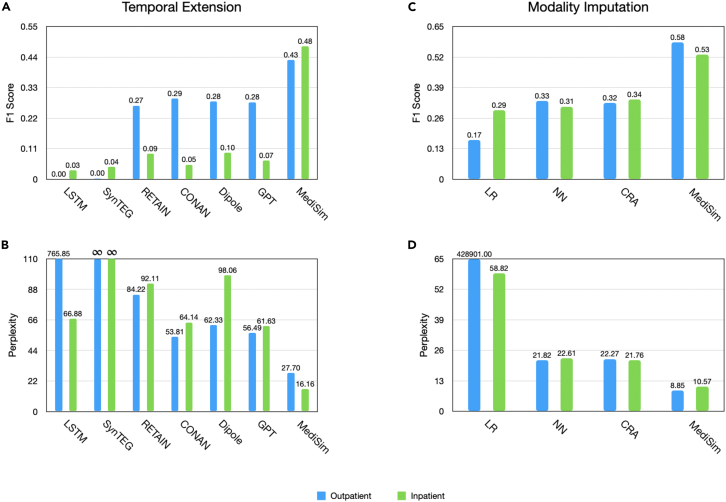
EHR simulation results comparing the abilities of different methods to predict medical codes Outpatient results are in blue, and inpatient results are in green. Note that MediSim is the only method which is capable of performing both types of simulation, and it achieves large performance gains on both largely through that flexibility. The results are (A) F1 score for temporal extension, (B) perplexity for temporal extension, (C) F1 score for modality imputation, and (d) perplexity for modality imputation.

We begin by assessing the ability of both MediSim and our baselines to predict medical codes based on prior visits. We make predictions for each patient in the test set. Each model’s predictions are evaluated as a binary classification task using the F1 score and as a probability distribution task using perplexity. The latter measures the test set’s likelihood, normalized by the number of present medical codes, indicating how well the compared methods model the distribution of patient records as a whole. Higher F1 scores and lower perplexity values are better. We show the results of these evaluations in Figure 2. The first takeaway is that only the MediSim can perform both temporal and modality simulation, offering flexibility, unlike any of the baseline models.

#### Temporal extension

We begin by comparing MediSim and the temporal extension baseline models on their ability to predict each of over 10,000 possible medical codes (i.e., diagnosis ICD [*International Classification of Diseases*], procedure Current Procedural Terminology [CPT], and medication NDC [National Drug Code] codes) based on prior visits. We see that MediSim outperforms each compared baseline in both metrics on both datasets. Specifically, while each of the stronger baselines such as RETAIN, Dipole, GPT, and CONAN perform similarly and fairly well on the outpatient dataset, MediSim can still achieve a 47% increase in F1 score, from 0.290 to 0.429 (as shown in Figure 2A), and a 48% decrease in perplexity, from 53.806 to 27.702 (as shown in Figure 2B), over the best baseline from that group, CONAN.

MediSim achieves the 47% improvement in either metric by using additional conditioning variables for each prediction, namely the previous codes in the same visit, that the temporal extension baseline architectures are not equipped to use despite their availability. This additional conditioning is used more explicitly in the modality simulation and completion, but its integration allows for a simultaneous and sweeping improvement in this temporal extension performance.

We observed that MediSim’s significant improvement over the baseline models in the inpatient EHR dataset is a perfect example of the value of this more flexible conditioning. While our stronger baselines (e.g., GPT, Dipole) for the outpatient dataset showed a much weaker performance across the board due to the more limited temporal nature of the inpatient dataset, meaning they had less to condition their predictions on, the dataset having more medical codes per visit in the inpatient dataset actually helped improve the performance of MediSim as it had additional conditioning variables and specifically ones that the baselines were not equipped to use. In fact, MediSim ’s F1 score had increased from 0.429 in outpatient settings to 0.479 in inpatient settings. As a result, MediSim achieved a 390% increase in F1 score from 0.095 to 0.479 and a 72% decrease in perplexity from 61.626 to 16.160 over the leading temporal extension baselines, GPT and Dipole, for the two metrics, respectively, in this inpatient setting.

We also show in Figure S3 that this impressive performance is relatively spread evenly across visit numbers, showing that it can be applied to perform effective simulation for any type of input data.

#### Modality imputation

Here, we discuss how well MediSim and the modality imputation baseline models can predict the presence of procedure (CPT) and medication (NDC) codes in each visit, based on the diagnosis codes (ICD) given in that visit and all previous visits. Note that the baseline models for modality imputation do not take time into account and only predict additional modality codes based on the current diagnosis codes. Figures 2C and 2D show that MediSim achieves better performance thanks to its more flexible and powerful architecture, which can condition each prediction on a much larger number of codes than any of the baselines.

The LR baseline performs poorly on both datasets, while the NN and CRA baselines perform similarly. In the outpatient setting, the NN performs slightly better than the CRA, while in the inpatient setting, the CRA performs slightly better than the NN. However, MediSim consistently produces better results in both outpatient and inpatient settings. It achieves a 74% improvement in F1 score, from 0.333 to 0.582 (as shown in Figure 2C), and a 59% decrease in perplexity, from 21.817 to 8.853 (as shown in Figure 2D), compared to the NN baseline in the outpatient setting. Similarly, it yields a 52% improvement in F1 score, from 0.339 to 0.530, and a 51% decrease in perplexity, from 21.760 to 10.566, compared to the CRA baseline in the inpatient setting.

#### Ablation results

One of our enhancements to the Hierarchical Autoregressive Language Model (HALO) architecture, beyond the data formatting and simulation flexibility to allow for modality imputation and the support for complex modalities, is using the self-supervised reinforcement learning training approach after the initial binary classification training. Due to the similarities between MediSim and HALO (it is designed as an extension and application of HALO, and in the temporal simulation setting where variable ordering does not matter, removing this self-supervised training makes HALO and an ablation identical), we avoided adding ablation results into Figure 2. However, we add them here to demonstrate the effect of this self-supervised training regime.

We compare the simulation performance of the full MediSim model with its ablation (which is the same as HALO in the temporal extension setting) in Table 1. There, we see a small but consistent improvement due to this additional training. Furthermore, we note that the improvement is more defined in the smaller data setting with the inpatient MIMIC dataset than in the extremely large outpatient dataset, which is in line with the motivation of the methodology.

**Table 1:** Temporal extension ablation performance

	Temporal extension				Modality extension			
	Outpatient EHR		Inpatient EHR		Outpatient EHR		Inpatient EHR	
	Perplexity	F1 score	Perplexity	F1 score	Perplexity	F1 score	Perplexity	F1 score
HALO/MediSim w/o self-supervision	27.729	0.428	16.312	0.466	8.875	0.580	10.655	0.516
MediSim	**27.702**	**0.429**	**16.160**	**0.479**	**8.853**	**0.582**	**10.566**	**0.530**

### Q2: Enhancing EHRs for predictive modeling

Real-world EHR data can be noisy and deficient in various ways. For instance, they may have temporal missingness, where some visits are missing, and modality missingness, where some types of medical codes are missing, such as procedures and medications. These deficiencies can negatively impact downstream tasks, such as building clinical predictive models for predicting specific disease phenotypes.

**Figure 3:**
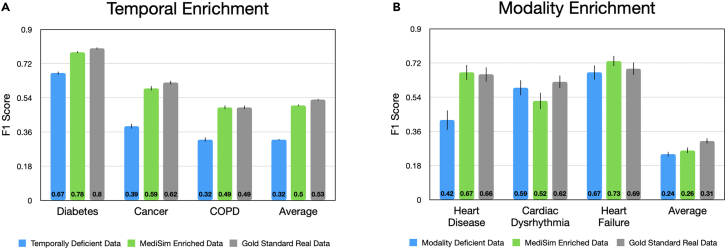
Downstream enrichment results showing the ability to improve the performance of predictive models by enriching deficient training data with MediSim We show (A) temporally enriched data compared to deficient, limited-length data and (B) modality-enriched data compared to deficient, diagnosis-only data. We see that MediSim allows downstream models to outperform their standard results on deficient data, especially in the temporal enrichment setting, and achieve results that approximate those of training on gold standard real data. Error bars refer to confidence intervals according to the standard error from bootstrapping the test sets 100 times.

In this experiment, our aim is to explore the effects of such missingness and assess whether generatively completed data can enhance the performance of deficient EHR datasets. This algorithmic augmentation provides comprehensive and full-modality datasets for training downstream machine learning prediction models. To achieve this, we use both forms of simulation and compare them against the original data on a series of downstream tasks. We focus here on this enhancement task itself comparing MediSim against deficient and gold standard real datasets, but we note that other baseline methods from the previous section can also be successful in performing this enhancement, albeit in more limited settings of a single type of missingness per baseline, and we provide a full set of enhancement results in Tables S3 and S4 for temporal and modality enrichment, respectively. Our findings are presented in Figure 3, where we also present the results by simulation type (temporal extension vs. modality imputation).

### Q2-a: Temporal enrichment

#### Setup

We start by examining the value of temporally extended data using the outpatient EHR dataset, which contains more visits per patient. We select 90,000 patients from the original outpatient test dataset for training and validation, and then 25,000 for testing. Note that we use the original test dataset even for training during this experiment to prevent data leakage and simulate the desired setting where our MediSim model is enriching an unseen deficient training dataset. To create our training dataset, we take the first 10 real visits for each of the new training patients as the real, temporally deficient dataset (and we explore the effect of this deficient record length hyperparameter in Figure S5). We then use MediSim to simulate additional visits for each record (we show in Figure S2 that this produces records that closely match the distribution of lengths in the original data). With the three different training datasets—(1) the real, temporally deficient data, (2) the augmented temporally enriched dataset, and (3) the real, full-length data (which serves as a gold standard dataset to provide a theoretical performance ceiling)—we train different machine learning models for various tasks. Specifically, we train prediction models for each of the 11 patient chronic condition categories used by the Centers for Medicare & Medicaid Services’ SynPUF synthetic dataset,^22^ with the goal of predicting the corresponding diagnosis codes in the next five unseen visits. We also provide Figure S1 to make the relationship and construction of these three types of datasets clearer.

#### Results

We present the test results for the three most common categories and the average across all 10 categories with a non-zero F1 score on the upper-bound real, full-length data in Figure 3A. The simulated data show an average 58% improvement in performance compared to the real but deficient limited-length data, increasing the mean F1 score from 0.316 to 0.498, where this score of 0.498 also very closely approximates the gold standard performance of real, full-length data. This overall enhancement is significant and also consistent, with clear improvements in each of the 10 tasks. Similarly, consistent improvements are also achieved by most of our compared temporal simulation methods within Table S3 in our supplement, where the GPT baseline slightly outperforms MediSim. Therefore, we demonstrate the significant value of this temporal simulation and enrichment approach.

### Q2-b: Modality enrichment

Next, we perform similar experiments and reach similar findings with modality-extended data on the inpatient EHR dataset.

#### Setup

We selected 7,000 patients from the inpatient test dataset for training and validation and 2,000 for testing. We started with the real, full-length records as our new inpatient training data but included only the diagnosis codes for each visit. We then expanded these records by adding simulated procedures and medications to each time step. We created three versions of the training datasets for comparison: (1) the modality-extended datasets, (2) the real diagnosis-only data, and (3) the real, full data (as another theoretical ceiling on performance).

Note that we assume the presence of diagnoses due to their central importance among medical codes, their overall prevalence, and their direct labeling capabilities, but this is largely used to set up this particular experimental setting. This experiment (and all of the modality-simulation results) could be repeated using different presence assumptions and thus different code modality orderings to similar effect. We address further implications and possibilities of this nuance within our discussion.

We trained disease prediction models on each of the new datasets for 25 different disease categories following the popular MIMIC-III benchmark setup.^23^ The prediction labels are defined by having the corresponding diagnosis codes in the next unseen visit, so to emphasize the effect of high-quality procedure and medication codes and prevent reliance on just the diagnoses, we added noise to the diagnosis codes by randomly replacing one code in each visit with another random code in the vocabulary (and we explore the effect of this noise hyperparameter in Figure S6). We presented the test set results for the tasks corresponding to the three most prevalent disease phenotypes, as well as the average overall tasks with nonzero F1 score on the upper-bound real, full-modality data (omitting eight extremely low prevalence phenotypes).

#### Results

Figure 3B shows a clear benefit from modality enrichment. MediSim offers a 7% boost over the modality deficient real data, from 0.244 mean F1 score to 0.262 averaged across all diseases. We also listed the top three most prevalent target conditions (heart disease, cardiac dysrhythmia, and heart failure). We also provide a comprehensive set of results, including simulation from all baselines and for all tasks in Table S4. The results indicate that while the baselines show limited performance in the temporal enrichment experiments, this can be attributed to the complexities of the task, particularly with a smaller dataset and sparse labels. However, MediSim demonstrates promising potential, especially in tackling the challenging rare disease labels. It outperforms the deficient data on 6 out of the 8 most prevalent tasks, achieving an impressive 14% increase in average F1 score from 0.421 to 0.478. This accomplishment highlights MediSim’s ability to excel in demanding scenarios and emphasizes its efficacy in the modality enrichment task.

### Q3: Handling complex modalities

We now transition toward evaluating the ability of MediSim to handle more complex modalities such as clinical notes and medical images and showcase not only that it can generate missing samples of these data points but moreover that it can use those generations or the modalities themselves to enhance both its predictions and the predictions of downstream models. To this end, we build two additional multimodal datasets, expanding on our original MIMIC inpatient EHR dataset.^17^ For the clinical notes modality, we find 41,103 patient records with 1.27 visits per record, each accompanied by a discharge note. For the imaging modality, we then use the MIMIC-CXR-JPG dataset,^18^ which contains longitudinal patient records with chest X-rays and derived structured findings at each visit. This dataset has 65,374 patient records with 2.90 visits per record, each defined by a 256 × 256 grayscale chest radiography image and 14 variables corresponding to positive, negative, unknown, or missing for each structured finding. We provide more details of these modalities in the supplement.

#### Generating complex modalities

**Figure 4:**
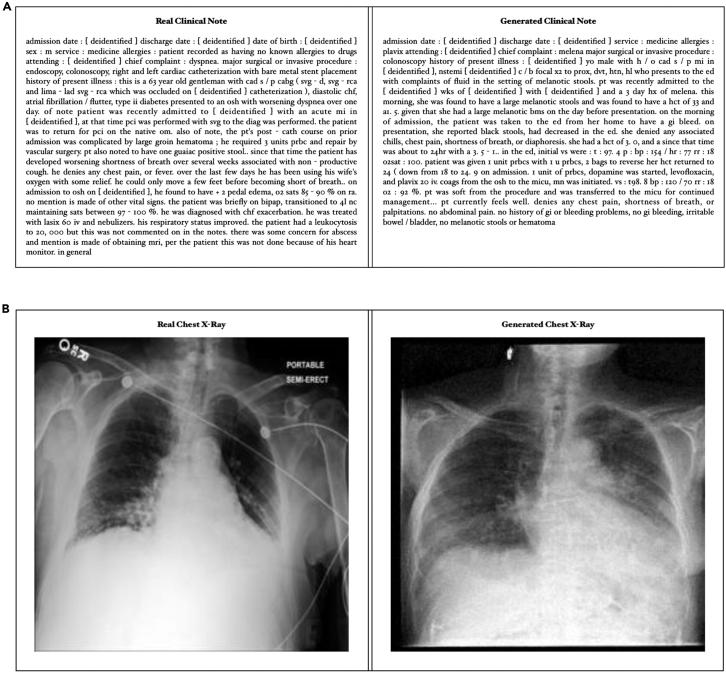
MediSim-generated samples of complex medical data modalities (Top) A synthetically generated clinical note and the real clinical note from the same visit. (Bottom) A synthetically generated chest X-ray and the real image from the same visit. While the generative models act as a sample approach to demonstrate support within the MediSim framework rather than fully optimized models, they still show the ability to approximate the format and content of the real data and also incorporate the conditioning content, such as the presence of a colonoscopy in the note and lung opacity in the image.

Using these datasets, we train conditional note and image generation models to demonstrate the ability of MediSim to translate structured information to simulated unstructured modalities. A sample generation from either model can be seen in Figure 4, with additional samples provided in Figure S7 for notes and in Figure S8 for images.

Figure 4 (top) shows a comparison between a randomly generated synthetic note (created with a sampling temperature of 0.75) for a visit in the test set, and the actual note from the same structured visit. The note generator is able to capture overall patterns in the formatting and content of discharge notes from the dataset. Additionally, the synthetic note includes some details from the structured medical codes, accurately capturing the service of the visit (medical allergies) and the procedures performed (colonoscopy) in this specific example. This synthetic note can be used for privacy-preserving data analysis or machine learning tasks, or with further improvement could serve as a substitute for a real note in enriching structured data where unstructured modalities are not available. It is worth noting that while there are clear gaps between the notes, such as incorrect age, gender, and a number of grammatical mistakes, we note that these derive from a pair of sources. First, the underlying data representation, which is shared from earlier experiments, did not include demographic information or a number of other details such as labs. Second, the model is meant more as a small, locally trained proof of support for complex modalities within the MediSim framework for complex modality support rather than a fully optimized text-generation model to be used in real-world settings. Therefore, the performance and quality of synthetic notes (and images later) could be improved further by optimizing the model selection and training as well as conditioning the generation with additional structured information such as demographics, labs, and visit outcomes, rather than just the visit vector containing diagnoses, procedures, and medications.

Figure 4 (bottom) demonstrates MediSim’s image-generation capabilities by comparing a randomly generated synthetic image for a visit in the test set to the true image from the same structured findings. In this comparison, we can see that the MediSim image generator is not only able to produce a realistic-looking X-ray overall but it can also effectively incorporate structured medical information to include lung opacity in the image.

#### Enhancing complex modality datasets

Next, we show that the data generated by our MediSim framework can enhance the predictions of machine learning models. We create synthetic versions of our training and validation clinical note datasets based on the actual structured codes. Subsequently, we train various classification models to forecast the presence of each condition code variable using the discharge note. The different models are developed using different training data.

(1) Full-real model: trained on the complete real training and validation datasets
(2) Deficient-real model: trained on a random 25% subset of the real data to simulate real-world missingness
(3) Synthetic model: trained on the complete synthetic training and validation datasets
(4) Real+synthetic model: trained on both the real and synthetic data

We then test each model on its ability to predict the top 25 most common codes in the real test dataset, and we show the results in Table 2. We note that the synthetic model performs very similarly to the full-real model (0.520 vs. 0.582 F1 score), while the real+synthetic model performs even better than the full-real model (0.602 vs. 0.582 F1 score).

**Table 2:** Discharge note classification performance showing that MediSim’s generative enhancement works in complex modality settings as well through the improvement of downstream clinical note classification models via the imputation of missing notes and the augmentation of additional notes even beyond the gold standard data

Training data	F1 score	AUROC
Full-real	0.582	0.829
Deficient-real	0.244	0.492
Synthetic	0.520	0.817
Real+synthetic	0.602	0.836

In summary, MediSim’s synthetic data can closely replicate the performance of the base model trained on the full real dataset. This capability allows for the approximation of real data and performance in the presence of real-world data limitations, such as those caused by privacy concerns. Additionally, we observe that enhancing the real dataset with synthetic data leads to improved performance, even though no additional real data were introduced into the pipeline, including during the training of the MediSim note-generation model.

#### Incorporating complex modalities

Finally, we show that we are also able to leverage real data from these modalities to improve MediSim’s core structured simulation. Therefore, for our multimodal chest X-ray dataset, we train and compare a standard MediSim architecture ignoring the image modality, operating only on the structured data, against an enhanced MediSim architecture with the image embedding model included.

We compared the performance of the image-enhanced model to the standard architecture, focusing only on visits after the first hospital stay, where there is an image to condition on. The results are presented in Table 3. We observed a clear improvement in both temporal and modality prediction by leveraging the information in the X-ray images, leading to a reduction of approximately 5% in perplexity values. Hence, MediSim is successful in generating realistic synthetic data from additional modalities, creating useful training data in those modalities, and using those modalities to further enhance its structured simulation. We also offer similar results and findings with note-enhanced simulation performance in Table S6, albeit to a lesser degree due to the large number of less-relevant structured codes.

**Table 3:** Image-enhanced simulation performance demonstrating that MediSim can utilize the presence of imaging modalities within EHR data to improve the simulation of its structured medical codes

	Temporal extension		Modality extension	
	Perplexity	F1 score	Perplexity	F1 score
MediSim	2.428	0.819	1.992	0.865
MediSim+images	**2.274**	**0.836**	**1.894**	**0.878**

## Discussion

In our paper, we address the issue of limitations in EHR data due to data quality issues, such as limited longitudinal information and missing modalities commonly found in real-world health datasets. We propose to solve this problem through EHR enhancement, handling any deficiencies by generatively extending health records along both the temporal dimension and the visit dimension. This involves adding new, simulated visits and adding new, simulated modalities of medical codes, clinical notes, medical images, and more. Our proposed method, MediSim, is differentiated from baseline methods in its ability to solve both extension problems simultaneously. It is specifically tailored to handle the sequential, multi-granular setting of EHRs. We have demonstrated that this method can achieve state-of-the-art performance in simulating medical records and producing enriched data that is more effective for training downstream machine learning models. We believe that our task and its solution offer an exciting approach to overcoming real-world health data deficiencies and facilitating improved machine learning research in this domain.

For example, patients often receive care from multiple healthcare providers who utilize independent record-keeping systems, making data integration challenging or even impossible. Consequently, the patient data available at any given healthcare facility frequently lack comprehensive documentation of previous visits, prescribed medications, or clinical notes generated elsewhere. MediSim alleviates these common real-world scenarios by simulating the missing visits and associated modalities, including absent medication records and clinical notes, thereby generating a more complete representation of a patient’s medical history. While these simulated records are inherently synthetic approximations rather than exact reproductions, they closely reflect realistic patient patterns, enabling healthcare institutions to enhance their existing datasets. Such enriched data can significantly improve downstream clinical tasks, such as disease classification and readmission prediction, ultimately supporting more effective and reliable medical machine-learning models.

### Limitations of the study

Despite the overall effectiveness of the simulation and the positive downstream impact, there are a number of assumptions and underlying limitations to the experimental setup that warrant discussion and pose interesting future work. First, there remains a requirement of high-quality training data that approximates the target or expected downstream distribution. MediSim is able to extend subsequent low-quality training data to closely match this distribution even in the face of huge encountered deficiencies, but a gap between MediSim’s training dataset and the ultimate downstream testing dataset will yield a disconnect. MediSim’s reinforcement learning-based training aims to help limit the reliance on this initial high-quality training data being overly large, but future work exploring how to further limit this need or introduce a tuning stage to allow the initial training on low- or mixed-quality data would be valuable.

Additionally, a difference in generation quality for different subgroups could have a negative downstream impact. While this is a challenge to analyze due to many different possible group definitions, it is an extremely important nuance to be aware of as with all machine learning. We explore two possible definitions in our supplemental information, where we show that MediSim does not disturb performance grouped by number of visits relative to real, high-quality downstream training data in Figure S4, but it does enhance the downstream performance of more prevalent disease prediction tasks to a greater degree as compared to rarer conditions on the inpatient dataset in Table S4. Therefore, further analysis of such impacts and attempts to identify and mitigate any bias make for important future work.

### Additional modalities

In this work, we demonstrate MediSim’s handling of structured EHR, clinical notes, and medical images; our approach can easily expand to handle and simulate any type of medical data, including lab time series (such as are found within a hospital stay), genomic information, and waveform data. Additionally, setups for clinical notes and imaging modalities can be duplicated to support various types of text and images, such as discharge notes, radiology reports, X-rays, and MRI images. Any combination or subset of these fields can be included in the same way, with generator and embedder models tailored based on specific use cases and available training data. These models can be configured independently, with each generating based only on the structured information, or autoregressively, with each conditioning their generation on the structured embedding of the previous additional modalities. This approach greatly enriches and enhances existing patient data by simulating missing visits and modalities, simulating a comprehensive ecosystem of complete patient records. However, as we noted in our discussion of the results of the complex modality generation, the quality and fidelity of this synthesis are a result of both the power of the decoder model and its training process, as well as the inclusion of all relevant variables in the conditioning information, as anything not provided cannot be expected to be used. Thus, the primary goal of these models in the context of the MediSim framework is the enhancement of training datasets for better downstream model training as opposed to the creation of precise clinical documentation.

### Code modality orderings

Finally, we note that within our data definition and experiments, we order the structured code modalities according to diagnosis, then procedure, and then medication. This ordering yields a generation process in which procedure generation is conditioned on diagnoses and medication generation is conditioned on both diagnoses and procedures. We made this choice due to the centrality of diagnoses within health care in terms of importance, prevalence, and labeling. However, we can certainly explore a different ordering for different applications. The ordering (both of code modalities and of codes within those modalities) could be further optimized. Additionally, a suite of models, each possessing its own ordering, could be trained and used interchangeably to maximize the data that can be conditioned in any given case of missingness. To demonstrate this interchangeability, we include prediction quality metrics similar to those used for Q1 for a new MediSim model trained and tested with a medication-diagnosis-procedure ordering in Table S5 in our supplement. This introduces a number of exciting questions and possibilities in terms of building and using such a mixed model, which we leave to future work.

## Methods

Our study acquired exempt status from the institutional review board pursuant to 45CFR46.104(d)(4): “Secondary research for which consent is not required: Secondary research uses of identifiable private information, if (i) The identifiable private information is publicly available; And (ii) Information is recorded by the investigator in such a manner that the identity of the human subjects cannot readily be ascertained directly or through identifiers linked to the subjects, the investigator does not contact the subjects, and the investigator will not re-identify subjects.”

### Background and related work

#### Patient data generation

Several works in patient data generation have focused on using GANs to create a paired architecture that undergoes adversarial training to generate realistic synthetic data.^4^^,^^15^^,^^24^^,^^25^^,^^26^^,^^27^^,^^28^ Another popular method for generating EHR data is the use of VAEs, as exemplified by EHR variational autoencoder (EVA).^16^ However, these methods generate EHR sequences all at once from a single latent representation, meaning they are unable to extend or complete real records.

Fortunately, two methods in this category can be adapted to provide at least a partial solution for our problem. The first one is SynTEG,^15^ a GAN-based method that generates individual visits conditioned on a representation of all past visits. This method is capable of conditioning on real visits and extending them with new, synthetic visits. However, it is not an explicitly predictive or probabilistic model, and it is still not capable of extending data within real visits to simulate missing modalities.

The second one is HALO,^29^ a language model-style architecture that models a probability distribution using visit- and code-level structures. However, it has a pair of limitations: it cannot generate complex modalities such as clinical note text, and it has a lack of structure within its data representation along with insufficient flexibility in its sampling technique to separate out the generation of different output code modalities. Therefore, in this paper, MediSim adapts and extends the HALO model to handle multi-modal data and bolster robustness with limited training data.

#### Medical code prediction

Our work is closely related to medical code prediction, which aims to predict codes for future visits based on previous medical records. There are several predictive models like GRAM,^2^ RETAIN,^3^ and Dipole^5^ that can predict a single label over a flexible future duration, but they can also be adapted to predict each code in the upcoming visit. Sequential models, such as recurrent NNs (RNNs), or language models, such as GPT,^19^ can also be adapted to the healthcare domain to perform the same task. However, these approaches may not be suitable for datasets that have missing modalities.

#### Handling missing patient data

Dealing with incomplete or missing data can be challenging, especially when entire data modalities are missing. Most of the existing literature focuses on imputing missing individual binary, discrete, or continuous values. These techniques include simple statistical methods such as mean imputation,^30^ k-nearest neighbor-based imputation,^31^ multiple imputations,^32^ and expectation maximization.^33^ This is also the case within the medical domain, where a large number of works explore the EHR data-quality issue but focus on imputing individual missing values^34^^,^^35^^,^^36^^,^^37^ and whether it is the best approach.^38^ However, these missing data techniques are often not enough to handle entirely missing data modalities, resulting in large, contiguous chunks of missing input values. Some methods can handle missing data modalities, but they still rely on data imputation, which involves reconstructing the embeddings or inputs of the missing data based on present ones.^21^^,^^39^^,^^40^^,^^41^^,^^42^^,^^43^ While these techniques can be adapted to address our second sub-problem of modality extension, none of them are designed to tackle temporal missingness.

#### Incorporating additional modalities

There have been various attempts to generate unstructured and complex medical modalities. However, none of these works have incorporated them into a comprehensive EHR generation and completion framework. These works usually focus on one or two modalities only and cannot handle multi-modal data directly. Our goal is to utilize these works to generate clinical note text^44^^,^^45^^,^^46^^,^^47^ and medical images^48^^,^^49^^,^^50^^,^^51^ in the context of other structured EHR fields. This will help us simulate complete and comprehensive patient data.

### Experimental setup

We provide more complete experimental details here for full contextualization and reproducibility of our results.

#### Data

Our two datasets are an outpatient EHR dataset consisting of IQVIA’s proprietary real-world US claims data and an inpatient EHR dataset from the public MIMIC-III ICU stay data.^17^ The outpatient EHR data represent a 5-year snapshot from 2013 to 2018 of IQVIA’s PharMetrics Plus for MedTech dataset, a longitudinal database of medical and pharmacy claims that spans the entire United States, contains over 200 million people in total, and has been estimated to cover 40% of all US claims,^52^ largely stemming from commercial health plans. Our subset contains roughly half a million different patients. The inpatient EHR data then contain just over 46,000 different patients who were admitted to the Beth Israel Deaconess Medical Center between 2001 and 2012. The outpatient EHR data are significantly more longitudinal, while the inpatient EHR data contain significantly more procedure and medication codes, and we present statistics quantifying these characteristics among others in Table S2. Finally, we keep all records, limit the records to a maximum of 100 visits, and perform no additional preprocessing on the contained medical codes.

#### Baselines

We consider the following baseline models, outlined in more detail than in our results section.

#### Temporal extension

(1) GPT^19^ consists of 12 transformer decoder blocks in GPT augmented to support multi-hot as opposed to one-hot inputs and outputs within the embedding layer and final activation layer.
(2) LSTM^20^ is a deep autoregressive LSTM model and is directly analogous to the GPT baseline but using blocks of LSTM recurrent neural networks instead of transformer decoder blocks.
(3) SynTEG^15^ is a GAN-based model that uses a transformer and LSTM-based encoder model to generate embeddings of EHRs up to a given visit before feeding those embeddings into a conditional GAN, which generates the next visit.
(4) RETAIN^3^ is a dual-attention-based health risk prediction model and is adapted here to operate in the forward rather than the backward temporal direction and to predict each code in the next visit.
(5) CONAN^4^ is an attention-based predictive healthcare model that is adapted here to convert the RNN to be unidirectional rather than bidirectional to maintain the autoregressive property and to predict each code in the next visit.
(6) Dipole^5^ is an RNN-based predictive healthcare model that is adapted here to predict each code in the next visit.

#### Modality extension

(1) LR is implemented to predict the missing modalities based on the medical codes in the present modalities.
(2) NN is a feedforward NN architecture mirroring the logistic regression approach of predicting the missing modalities based on the medical codes in the present modalities, but with two hidden layers of sizes 256 and 128 before the final prediction layer.
(3) CRA^21^ is a missing modality imputation method that reconstructs a full output from a partially missing input through a series of autoencoder networks that predicts the difference between their input and the true output.

#### Evaluation strategy

We use a 72-8-20 train-validation-test split for both datasets. We use the Adam optimizer with a learning rate of 1e−4, batch size of 48, and train for 50 epochs on a single NVIDIA Tesla V100 GPU. For the reinforcement learning-based training, we set a maximum number of iterations of 10 (although we never reach that maximum). Finally, we implement the model and train using the PyTorch framework.

#### Metrics

We assess our models based on predictive accuracy and enriched data utility, using two key metrics: F1 score for binary prediction and perplexity.

First, we use the F1 score as defined as follows:

(Equation 2) $$F1=\frac{2*Precision*Recall}{Precision+Recall}=\frac{2*TP}{2*TP+FP+FN}$$

where TP, FP, and FN represent true positives, false positives, and false negatives, respectively.

Second, we also use perplexity for predictive modeling evaluations. It measures the test set’s likelihood as determined by a model trained on the training set, normalized by the number of medical codes in a patient’s record. Mathematically, it is defined as

(Equation 3) $$\begin{array}{c} PP\left(D\right)={\left(P\left(D\right)\right)}^{-1/N}\\ ={\left(P\left(R^{\left(1\right)},\ldots ,R^{\left(\left|D\right|\right)}\right)\right)}^{-1/N}\\ ={\left(P\left(R^{\left(1\right)}\right)\cdots P\left(R^{\left(\left|D\right|\right)}\right)\right)}^{-1/N} \end{array}$$

where D is the test dataset, $R^{\left(i\right)}$ is the i-th record in D, and N is the total number of medical codes in D (roughly 2 million in our inpatient dataset and over 80 million in our outpatient dataset). In practice, we calculate the values by summing their log probabilities, using the equivalent form

(Equation 4) $$PP\left(D\right)=\exp\left(-\frac{1}{N}\sum_{R\in D}\log\;P\left(R\right)\right)$$

The normalized value then also corresponds to how many of the different normalizing units (medical codes) one would have to randomly pick between on average to achieve the same probability.

For downstream performance evaluation, we primarily use the F1 score while also including the typical binary classification metric area under the receiver operating characteristic curve (AUROC) in some experiments.

### Terminology and data formatting

We provide additional details regarding our data and problem formulation, including how it is converted to a structured format for machine learning model input.

#### EHR data

We represent a patient record R as a sequence of visits over time as given by Equation 1, where each visit $V^{\left(t\right)}$ is composed of a varying number of medical codes and other medical modalities as given by the equation in the section EHR deficiencies. To enable input to our MediSim model, we convert the structured medical codes from R (excluding any unstructured text, images, or other additional modalities for now) into a matrix representation R. This is achieved by constructing a matrix R comprising a sequence of vector representations $v_{1},v_{2},v_{3},\ldots ,v_{T}$ for each of the patient’s T visits. Each visit $v_{t}\in R^{\left|D\right|+\left|P\right|+\left|M\right|}$ is then represented as a multi-hot binary vector using ones to represent the medical codes present in that visit. Within that vector, $c_{t}^{i}$ represents the binary variable indicating the presence of the i-th code in the t-th visit of R. The codes are arranged into three regions, representing the modalities of diagnoses, procedures, and medications. Therefore, $c_{t}^{0}$ to $c_{t}^{\left|D\right|}$ indicate the presence of diagnosis codes, $c_{t}^{\left|D\right|}$ to $c_{t}^{\left|D\right|+\left|P\right|}$ indicate the presence of procedure codes, and $c_{t}^{\left|D\right|+\left|P\right|}$ to $c_{t}^{\left|D\right|+\left|P\right|+\left|M\right|}$ indicate the presence of medication codes.

#### Stop code for EHR generation

At the end of the record in $v_{T}$, we include a special last code to signal the end of the record. This code allows us to exclude any additional columns from R. This code is included as a variable in each visit vector. It enables a model to learn when to stop the record, similar to how a stop token allows language models to halt text generation. Additionally, it allows us to convert R into a longer standard length for batch input of multiple patients. We then approach our task of generating $R^{\prime }$ by generating $R^{\prime }$ through our MediSim model, which learns and samples from the probability distribution $P\left(R|R^{*}\right)$.

#### Handling other modalities

Finally, we describe our approach to handling complex modalities, such as clinical notes and medical images, that cannot be easily integrated into R. These modalities have valuable information beyond structured medical codes, but they are often neglected by machine learning EHR representations and existing predictive and generative models due to their complex or unstructured formats and privacy constraints. We outline a process to enable such methods to generate and handle these unstructured data modalities within the context of their existing structured data.

To generate unstructured modalities, we create a conditional probability from structured representation to unstructured modality. Specifically, these modalities can be conditionally generated based on the contents of the current visit by learning and sampling directly from $P\left(n^{\left(t\right)}|v_{t}\right),P\left(i^{\left(t\right)}|v_{t}\right)$, or other similar distributions to extend the task of modality simulation to the case of missing clinical notes, images, lab time series, and more.

Additionally, these modalities can be used as a part of a patient’s medical history to predict future structured visits by embedding them into a structured format. For example, a note $n^{\left(t\right)}$ can be embedded as $n_{t}\in R^{n_{n}}$, and an image $i^{\left(t\right)}$ can be embedded as $i_{t}$. These structured representations can then be appended to the end of each visit vector $v_{t}$, resulting in an enhanced visit representation $v_{t}^{\prime }$ with the same multi-hot binary representation as before, but with continuous, text, image, and other modality embeddings at the end. This process is formalized as

(Equation 5) $$\begin{array}{cc} n_{t} & =\text{emb}\left(n^{\left(t\right)}\right)\\ i_{t} & =\text{emb}\left(i^{\left(t\right)}\right)\\ v_{t}^{\prime } & =\text{concat}\left(v_{t},n_{t},i_{t}\right) \end{array}$$

In the context of patient data synthesis or augmentation, this enhanced visit and record representation can provide additional information used to simulate future structured medical codes. The structured codes can then be used to generate any additional modalities at each visit. These two operations can then also be combined for even better results.

We provide Table S1 containing all relevant notations for reference.

### MediSim

**Figure 5:**
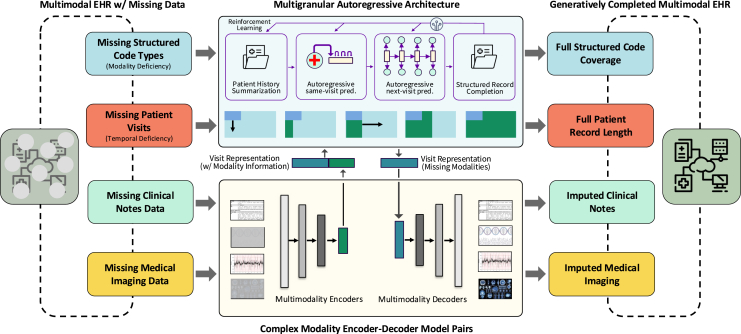
MediSim’s data enrichment framework MediSim is a temporal and multimodal simulation method that can extend and enrich real patient records both temporally and by simulating missing modalities. MediSim consists of a deep, multi-granular autoregressive simulation model and utilizes an iterative and reinforcement learning-based training regime, drawing inspiration from reinforcement learning from human feedback, to improve the performance of that architecture in low-data settings. Finally, it leverages encoder-decoder model pairs for handling and generating more complex modalities such as clinical notes and medical images.

MediSim is a patient data simulation method designed to fill in missing patient data, such as text, images, and other data modalities, while also expanding real patient records in both temporal and low-data settings. This method uses a deep, multi-granular autoregressive architecture to effectively simulate and expand patient data. The model is trained using an iterative and reinforcement learning-based regime to enhance its performance. Additionally, encoder-decoder model pairs are employed to handle and generate clinical notes, medical imaging data, and other complex data types. Figure 5 provides a visual representation of the entire process and model in MediSim.

#### Multi-granular autoregressive architecture

We model P(R), the distribution of longitudinal structured patient records R (e.g., diagnoses, procedures, and medications across multiple visits) by adapting a multi-granularity autoregressive architecture first proposed by Theodorou et al.^29^ This architecture can be broken down into two steps. First, based on a temporal subset of R containing visits $\left[v_{1},v_{2},\ldots ,v_{t}\right]$, the architecture summarizes or embeds the history of the patient through time step t as $h_{t}$. Second, based on that history, it autoregressively simulates the next visit $v_{t+1}$ one code at a time. These two steps combine to act as a “next-visit prediction” function that contains efficient patient representation while maintaining the intricate intra-visit modeling dependencies that allow for maximal performance and the ability to start at any point in the visit to simulate missing modalities of codes.

### Patient history summarization

The first module converts the sequence of patient visits $R=\left[v_{1},v_{2},\ldots ,v_{T}\right]$ into a sequence of patient history summaries $H=\left[h_{1},h_{2},\ldots ,h_{T}\right]$, where $H\in R^{T\times n_{\text{emb}}}$, and each summary $h_{t}$ represents the contents of all of the patient’s visits up through the t-th time step.

To achieve this, we use a stack of transformer decoder blocks. We pass each of the visit vector representations $\left[v_{1}\cdots v_{T}\right]$ through trainable positional and code-embedding matrices and sum the results to embed them each in $R^{n_{\text{emb}}}$. We then pass the visit embeddings through M=12 transformer decoder blocks, which use masked multi-head self-attention to generate a sequence of visit history representations that summarize the contents of all the visits up through a given point in the patient’s history. This module takes in only R and is written formally as

(Equation 6) $$\begin{array}{l} H^{\left(0\right)}=RW_{e}+W_{p}\\ H^{\left(m\right)}=\text{transformer}\_\text{block}\left(H^{\left(m-1\right)}\right)\;\forall m\in \left[1,M\right] \end{array}$$

where $W_{e}\in R^{\left(\left|D\right|+\left|P\right|+\left|M\right|+1\right)\times n_{\text{emb}}}$ is the code embedding matrix and $W_{p}\in R^{T\times n_{\text{emb}}}$ is the position embedding matrix (that adds a learnable embedding to each visit embedding based on its time step, a necessary calculation to recapture the temporal ordering information from the sequence of visits that transformers otherwise lose), and each transformer block is based on a decoder block from the original transformer architecture^53^ defined by

(Equation 7) $$\begin{array}{l} H_{1}^{\left(m\right)}=H^{\left(m-1\right)}+\text{Masked}\;\text{Multi}-\text{Head}\;\text{Self}-\text{Attention}\left(H^{\left(m-1\right)}\right)\\ H_{2}^{\left(m\right)}=\text{Layer}\;\text{Normalization}\left(H_{1}^{\left(m\right)}\right)\\ H_{3}^{\left(m\right)}=\left(H_{2}^{\left(m\right)}+\left(\max\left(0,H_{2}^{\left(m\right)}W^{\left(m\right)}+b^{\left(m\right)}\right)V^{\left(m\right)}+c^{\left(m\right)}\right)\right)\\ H^{\left(m\right)}=\text{Layer}\;\text{Normalization}\left(H_{3}^{\left(m\right)}\right) \end{array}$$

where $W^{\left(m\right)}$ and $V^{\left(m\right)}$ are the weight matrices in the feedforward portion of the m-th layer, and $b^{\left(m\right)}$ and $c^{\left(m\right)}$ are the corresponding bias vectors. The results of the final layer, $H^{\left(M\right)}$, are the module’s final output $H=\left[h_{1},h_{2},\ldots ,h_{T}\right]$, a series of patient history summaries after each of their visits.

### Autoregressive next visit prediction

These patient history representations are then used to predict the codes in the subsequent visit in the next module. Specifically, given any $h_{t}$, we aim to predict each code $c_{t+1}^{i}$ in $v_{t+1}$. However, instead of predicting the codes all at once, we break the prediction down into an autoregressive sequence where each code $c_{t+1}^{i}$ is predicted based on both $h_{t}$ and all of the previous codes in the sequence $c_{t+1}^{1},c_{t+1}^{2},\ldots ,c_{t+1}^{i-1}$. Therefore, we model the probability $P\left(c_{t+1}^{i}|h_{t},c_{t+1}^{1},c_{t+1}^{2},\ldots ,c_{t+1}^{i-1}\right)$ for each of the |D|+|P|+|M|+1 codes composing $v_{t+1}$.

To model the distribution of each $P\left(c_{t+1}^{i}\right)$, $h_{t}$ is fed, along with the partially completed next visit vector $v_{t+1}$ through N=2 masked linear layers, which maintain the same dimensionality and mask the inputs through an upper triangular weight matrix to ensure they preserve the autoregressive property (and have a rectified linear unit activation function between layers). The output corresponding to the i-th code (at index $n_{emb}+i$ when taking into account $h_{t}$) then corresponds to the predicted probability of $c_{t+1}^{i}$. This calculation is written formally as

(Equation 8) $$\begin{array}{c} h_{t+1}^{\prime \left(0\right)}=\text{concat}\;\left(h_{t},v_{t+1}\right)\\ h_{t+1}^{\prime \left(n\right)}=\text{masked}\_\text{linear}\left(h_{t+1}^{\prime \left(n-1\right)}\right)\forall n\in \left[1,N\right]\\ P\left(v_{t+1}\right)=\text{sigmoid}\left(h_{t+1}^{\prime \left(N\right)}\left[n_{\text{emb}}:\right]\right)\text{,} \end{array}$$

where the masked linear layers are achieved by

(Equation 9) $$\begin{array}{l} H^{\prime \left(n\right)}=\max\left(0,H^{\prime \left(n-1\right)}\left(W^{\left(n\right)}⊙M\right)+b^{\left(n\right)}\right)\text{,} \end{array}$$

where the max function is omitted for the final layer, ⊙ is element-wise matrix multiplication, $W^{\left(n\right)}$ and $b^{\left(n\right)}$ are the trainable parameters of the module, and M is the masking matrix, which is an upper triangular matrix of ones to preserve the autoregressive property.

#### Initial training

During the initial training phase, we feed entire records R through both the patient history summarization and autoregressive next visit prediction modules to generate probabilities for each code and visit at once. The probabilities are then compared with the actual presence of each code and used to train the network by backpropagating the corresponding binary cross-entropy loss function.

#### Patient simulation framework

While the architecture operates and generates a probability distribution over the entire record R, the actual sampling process is sequential, performed one code at a time. We feed the input record $R^{\prime }$, filled up through the i-th code in the t-th visit, into the model, generating the probability $P\left(c_{t}^{i+1}\right)$. We sample from this probability to produce $c_{t}^{i+1}$, which we add to $R^{\prime }$ before repeating the process for the next code.

Given the autoregressive format of MediSim’s predictions, it automatically has the ability to predict $P\left(R|R^{*}\right)$, regardless of the shape or content of $R^{*}$. Therefore, given a new deficient patient representation $R^{*}$, we can feed the limited record that we have into our model and sequentially sample from the corresponding probabilities to complete the missing values.

Specifically, we proceed left to right along the temporal dimension, stopping at any incomplete visits (including completely missing visits). For each incomplete visit $v_{t}^{*}$, we summarize the history of all previous visits with the first module, producing $h_{t-1}$. We then iterate sequentially over the visit vector with the second module, filling in any missing codes and variables we encounter as we go by randomly sampling from their predicted probabilities, producing $v_{t}$.

Therefore, if $R^{*}$ is longitudinally deficient, we can use the available visits to generate entirely new visits starting at $v_{T^{\prime }+1}$. If $R^{*}$ is modality deficient, we can create representations of visit history for each visit and then start sampling code-level probabilities after the diagnosis codes to account for the current modality and simulate the missing procedure and medication codes. If $R^{*}$ is deficient in both longitudinal and modality aspects, then we first complete the modalities of the present visits before generating entirely new visits from there. Figure 1 provides a visual representation of these three generation processes.

While we discuss the handling of additional complex modalities below, they are simply treated as the final data modality within this framework. Thus, for any visit where they are missing, once we have generated the full structured visit vector, we can use that to generate the unstructured modality’s data before proceeding to the next time step.

#### Iterative, reinforcement learning-based training regime

Our original architecture is trained using a binary cross-entropy loss function on all medical codes simultaneously, treating it as a large multi-label classification problem. To address the scarcity of rich, multimodal, longitudinal training datasets, we implement an iterative, reinforcement learning-based training regime inspired by reinforcement learning with human feedback. We substitute human rankings with a discriminator-style classification model that distinguishes real from synthetic records. This reward model refines the MediSim model to simulate realistic patients beyond dataset-learned probabilities while avoiding the infeasibility of propagating through the sequential generation process for direct GAN-based adversarial training, iterating through a cycle of MediSim model enhancement, synthetic data, reward model, and reinforcement learning training.

We first train the MediSim model as a classification task using all available data. Then, for each training iteration, we generate a synthetic dataset equal in size to the real training dataset using the current MediSim model, simulating synthetic records $R^{\prime }$ as if our deficient record $R^{*}$ was just the dummy “first visit.”

### Classification reward model

We train a weak RNN classifier to distinguish between real and synthetic patient records using a synthetic dataset, similar to training a GAN discriminator. The weak classifier avoids overfitting while capturing realism and coherence, especially for initial generations in low-data settings. We train the classifier using binary cross-entropy loss, with real records as positive labels. The trained classifier’s probability output serves as a proxy for record quality, acting as a value function $V:R^{\prime }\to v\in \left(0,1\right)$, mapping patient records to reward values between 0 and 1.

### Reinforcement learning training

Due to the sequential nature of the generation process, it is infeasible to directly backpropagate through this classifier and the generator as with the GAN objective. We instead use the value function to enhance our core MediSim model by optimizing it to maximize the expected reward, which is the predicted probability of being real records. We achieve this using the REINFORCE policy gradient algorithm.^54^

During each training step, we generate a batch of B synthetic patient records $\left\{R_{1}^{\prime },\ldots ,R_{B}^{\prime }\right\}$. Then, we calculate the probability and the reward for each generated record based on the simulation model. We use the mean log probability-reward product across the batch as the model’s current expected reward. We then perform gradient ascent on this value to increase the expected reward. The objective is to maximize the equation

(Equation 10) $$E_{R^{\prime }\in \left\{R_{1}^{\prime },\ldots ,R_{B}^{\prime }\right\}}\left[\log\left(P\left(R^{\prime }\right)\right)*V\left(R^{\prime }\right)\right]$$

After every v step, we evaluate the model’s performance on the validation set and save the model if it improves. This approach ensures that we remain focused on the original simulation task, using the reward model to better achieve our main objective, rather than overfitting to a secondary goal. Training continues until the model does not show any improvement for p consecutive steps.

We then move on to the next iteration, using the enhanced MediSim model to generate higher-quality synthetic data. This leads to a higher-quality reward model and better reinforcement learning training for further improvement. We repeat this process until we reach our maximum number of iterations or the model no longer shows improvement during a reinforcement learning training run.

#### Model pairs for handling additional modalities

In our MediSim method, we extend the core architecture to incorporate the use and generation of clinical note text, medical scan images, and other complex medical modalities.

### Generating clinical notes

We have explored various methods for learning $P\left(n^{\left(t\right)}|v_{t}\right)$ and generating clinical notes, including filling in templated text and utilizing large pre-trained language models like ChatGPT based on the descriptions of the medical codes present in the visit. Finally, we have chosen to train our own language model specifically for the task of conditioned clinical note generation to ensure that the generated notes closely match the format and content of the training dataset.

Specifically, we have trained a small, GPT-style^19^ transformer decoder generation architecture, $G_{n}$. We ensure that the text generated by $G_{n}$ accurately represents the content of the visit by replacing the typical start token with a learned embedding $v_{t}^{e}$ of the visit vector $v_{t}$, which conditions the rest of the text. This embedding is generated by feeding $v_{t}$ through a single trainable NN layer. At each step in token generation, G then predicts the next word or token based on the previous tokens as well as that embedding, sampling from $P\left(w_{t}|v_{t}^{e},w_{1},\ldots ,w_{t-1}\right)$, which it learns over the dataset in the typical way.

### Utilizing clinical notes

With the ability to generate clinical notes, we can now assume their presence in our EHR representation. To leverage them for enhancing patient modeling and simulating future structured medical codes, we embed the clinical note text and append the embedding to each visit vector, which can be used interchangeably with the fully binary visit vector within our core architecture. Instead of using pre-trained language models like ClinicalBERT, we train our own BERT-style transformer encoder embedding architecture, $E_{n}$, end-to-end with our core architecture. This allows the embedding to focus on additional details and predictive content relevant to our specific problem setting and dataset. We include a [CLS] token at the start of each clinical note and extract the final enriched class embeddings using $E_{n}$. These embeddings are concatenated to the structured record representation R before entering into Equation 6:

(Equation 11) $$\begin{array}{cc} N & =\left[E_{n}\left(n^{\left(1\right)}\right),E_{n}\left(n^{\left(2\right)}\right),\ldots ,E_{n}\left(n^{\left(T\right)}\right)\right]\\ H^{\left(0\right)} & =\text{concat}\left(R,N\right)W_{e}+W_{p} \end{array}$$

The only other adjustment is to change the shape of $W_{e}$ to handle the additional embedding dimensions in its new input.

### Handing imaging data

We use a similar setup with generator $G_{i}$ and embedder $E_{i}$ for generating and utilizing imaging data. While any pair of models and architectures can be chosen, we opt to train our own models for our specific task using our training data. Specifically, we train a diffusion model, $G_{i}$, which is conditioned on the learned embedding $v_{t}^{e}$. It is worth noting that this embedding may be shared or separate from other modalities. Additionally, we train a standard convolutional NN-based encoder model, $E_{i}$, alongside our core architecture by generating embeddings of each image, whether real or synthetic, and then concatenating them to the rest of the structured record representation before inputting it into our model.

### Generation process

After training the generator and embedding models, we can integrate additional modalities into our simulation process. We can follow the temporal dimension from left to right, simulating the history representation of all previous visits, including their additional modalities (embedded using $E_{n}$, $E_{i}$, and other models) for each visit. Then, we can proceed over the modality dimension. After completing the structured medical code modality simulation, if the clinical note and any other modalities are missing, we can generate them using $G_{n}$ and other corresponding generators along with the completed structured visit vector. This allows us to maintain the same flow and simulate completed and extended patient records, including additional complex, accompanying modalities we desire.

## Resource availability

### Lead contact

Requests for further information and resources should be directed to and will be fulfilled by the lead contact, Jimeng Sun (jimeng@illinois.edu).

### Materials availability

This study did not generate new materials.

### Data and code availability

• The MIMIC-III inpatient EHR dataset^17^ that we use is publicly available and may be downloaded and used freely after performing training and applying on physionet.org. The outpatient dataset is proprietary from IQVIA, but it may be licensed or accessed upon request.
• All original code has been deposited at Zenodo: https://doi.org/10.5281/zenodo.15058513 and is publicly available there as well as at https://github.com/btheodorou99/MediSim as of the data of publication.^55^ We further release each of the final trained MediSim models tested in this work at Zenodo: https://doi.org/10.5281/zenodo.13007242.^56^ Both the code and models are released under the Massachusetts Institute of Technology license.
• Any additional information required to reanalyze the data reported in this paper is available from the lead contact upon request.

## Acknowledgments

This work was supported by National Science Foundation award nos. SCH-2205289, SCH-2014438, and IIS-2034479.

## Author contributions

B.T. and J.S. proposed the method; B.T. conducted all the experiments; and B.T., C.X., J.S., and L.G. wrote the manuscript.

## Declaration of interests

The authors declare no competing interests.

## Supplemental information

Document S1. Supplemental methods, Figures S1–S8, and Tables S1–S6

Document S2. Article plus supplemental information
