# Supplementary material for: MediSim: Multi-granular simulation for enriching longitudinal, multi-modal electronic health records
Source: Patterns (N Y). 2025 May 8;6(6):101261. doi: 10.1016/j.patter.2025.101261 (PMC12191717; doi:10.1016/j.patter.2025.101261)
Supplement: Document S1. Supplemental methods, Figures S1–S8, and Tables S1–S6 [file mmc1.pdf]

Patterns, Volume *6*

Supplemental information

MediSim: Multi-granular simulation for enriching longitudinal, multi-modal electronic health records

Brandon Theodorou, Cao Xiao, Lucas Glass, and Jimeng Sun

Supplementary Material for "MediSim: Multi-Granular Simulation for Enriching Longitudinal, Multi-Modal Electronic Health Records"

Brandon Theodorou[1,2], Cao Xiao[2], Jimeng Sun[1,2*]
University of Illinois at Urbana-Champaign, 201 North Goodwin Avenue, Urbana, IL, USA[1]
Medisyn Inc., Las Vegas, NV, USA[2]
* To whom correspondence should be addressed: jimeng@illinois.edu

We aim for our main paper to be self-contained, comprehensive, and as straightforward as possible regarding our task, our proposed MediSim method, and its results. However, we add and discuss a few details and results here in greater nuance to offer further completeness.

1 SUPPLEMENTARY METHODS

1.1 Notation

We provide a table of notations for reference in Supplementary Table 1

1.2 Data Specification

We provide a series of statistics describing the size and shape of our two datasets in Supplementary Table 2.

1.3 Self-Supervised Training Hyperparameters

In our method section, we describe that we evaluate our model every v steps of reward model training and conclude a self-supervised iteration when a model fails to improve for p consecutive checks. We find a limited effect from changing either of these hyperparameters in our experiments, and we use $v = 10$ and $p = 5$ throughout.

1.4 Optimizing Generation Speed

An EHR consists of an extremely long binary sequence such that generating missing codes and visits would be an extremely slow process if a new probability distribution needed to be generated at each step. One of the values provided by MediSim is in grouping past visit representations through the visit-level module to allow processing this long sequence with full autoregressive dependencies while maintaining the nuances of intravisit, code-level modeling. However, this efficient representation can not avoid the long, code-by-code generation process. Luckily, the records are also very sparse, with the order of tens of medical codes selected out of thousands or tens of thousands at each visit. So, we can speed up generation by generating probabilities for all of the rest of the codes in the current visit assuming that they are all zero and sampling from all of the probabilities simultaneously. We then find the next positively sampled code and skip directly to it in the generation process by noting that all previous codes will remain zero, so its probability is still correct. In this way, we can also group code generations to speed significantly up generation.

Supplementary Table 1: Table of Notations

Notation	Description
$\mathcal{R}$	A patient's EHR medical record
$\mathcal{V}^{(t)}$	The t-th visit in $\mathcal{R}$
$d_i^{(t)}$	The i-th diagnosis code in $\mathcal{V}^{(t)}$
$p_i^{(t)}$	The i-th procedure code in $\mathcal{V}^{(t)}$
$m_i^{(t)}$	The i-th medication code in $\mathcal{V}^{(t)}$
$n^{(t)}$	The clinical note text in $\mathcal{V}^{(t)}$
$i^{(t)}$	The imaging scan in $\mathcal{V}^{(t)}$
$T \in \mathbb{N}$	The number of visits in $\mathcal{R}$
$\mathcal{D}$	The set of all diagnosis codes
$\mathcal{P}$	The set of all procedure codes
$\mathcal{M}$	The set of all medication codes
$\mathbf{R} \in \mathbb{R}^{T \times (\lvert\mathcal{D}\rvert + \lvert\mathcal{P}\rvert + \lvert\mathcal{M}\rvert + 1)}$	The matrix representation of $\mathcal{R}$
$\mathbf{v}_t \in \mathbb{R}^{(\lvert\mathcal{D}\rvert + \lvert\mathcal{P}\rvert + \lvert\mathcal{M}\rvert + 1)}$	The vector representation of the t-th visit in $\mathbf{R}$
$c_t^i \in \{0, 1\}$	The binary presence of the i-th code in $\mathbf{v}_t$
$\mathbf{n}_t \in \mathbb{R}^{n_n}$	The structured embedding of $n^{(t)}$
$\mathbf{i}_t \in \mathbb{R}^{n_i}$	The structured embedding of $i^{(t)}$
$\mathbf{R}_l \in \mathbb{R}^{T' \times (\lvert\mathcal{D}\rvert + \lvert\mathcal{P}\rvert + \lvert\mathcal{M}\rvert + 1)}$	A longitudinally deficient record
$\mathbf{R}_m \in \mathbb{R}^{T \times (\lvert\mathcal{D}\rvert + 1)}$	A modality deficient record
$\mathbf{R}_{l/m} \in \mathbb{R}^{T' \times (\lvert\mathcal{D}\rvert + 1)}$	A longitudinally and modality deficient record
$\mathbf{R}^* \in \{\mathbf{R}_l, \mathbf{R}_m, \mathbf{R}_{l/m}\}$	A global notation for any deficient record

Supplementary Table 2: Final Dataset Statistics

	Outpatient Data	Inpatient Data
Number of Records	595,498	46,520
Mean Visits Per Record	38.34	1.26
Mean Codes Per Visit	3.52	47.62
Mean Diagnoses Per Visit	2.42	11.04
Mean Procedures Per Visit	0.94	3.88
Mean Medications Per Visit	0.01	32.70
Unique Medical Codes	18,114	13,219
Unique Diagnosis Codes	11,226	6,984
Unique Procedure Codes	5,179	2,032
Unique Medication Codes	1,709	4,203

1.5 Temporal Enrichment Datasets

Within the experiments in our main paper, we examined the value of simulation in enriching datasets by adding additional visits or modalities to make the data more effective for training downstream predictive models. To do this, we compared three types of datasets. For each patient in the training data pool for this experiment, we took the first 10 real visits as the real, temporally deficient dataset. We then completed the data with either the real follow-up visits or those simulated using one of our compared methods. This yielded three different types of training datasets: 1) the real, temporally deficient data, 2) the augmented temporally enriched dataset, and

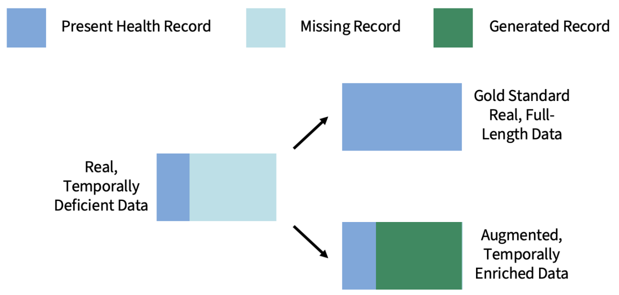

Supplementary Figure 1: The three different data setups for the temporal enrichment experiments. All begin with the same first 10 visits, but we then add real or simulated data to create two additional, enriched datasets to compare added utility for downstream predictive model training.

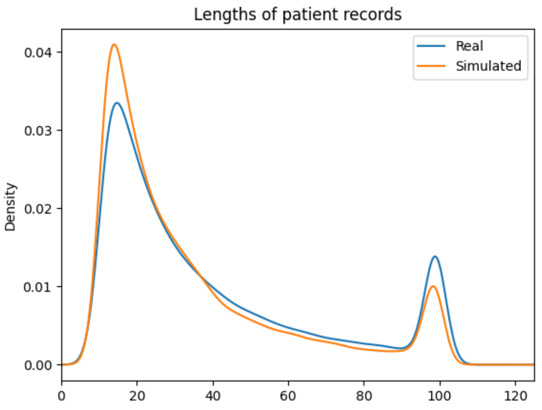

Supplementary Figure 2: The probability density of different record lengths in the real, full-length and `MediSim` extended datasets. The distributions demonstrate the variability in record lengths but also that `MediSim` accurately captures the patterns found in the real data.

3) the real, full-length data (which serves as a gold standard dataset to provide a theoretical performance ceiling) whose relationships are depicted in Supplementary Figure 1 to provide further clarity. However, we also aim to clarify that this process should be viewed as "completing" the deficient records rather than simulating a certain number of time steps. The simulation models can generate a "stop code" to cease generation, and both the temporally enriched and gold standard, full-length datasets vary in the number of visits. Our aim is that the simulated data closely matches the shape of the real data to avoid introducing any unnecessary biases, as discussed in our limitations within our main paper, so we demonstrate the distribution of the number of visits in `MediSim`'s simulated data and the original full-length data in Figure 2.

1.6 Imaging Dataset Modalities

The medical imaging EHR dataset we used [1] has structured information corresponding to various findings that may be derived from chest X-ray images. Specifically, it has 56 code variables, with 4 variables signifying positive, negative, unknown, and missing for each of the 14 possible findings. However, these variables are not readily grouped into diagnoses, procedures, and medications like the main EHR datasets. So we define the following four new modalities grouped by type of finding:

- Cardiopulmonary Conditions
  - Cardiomegaly
  - Edema
  - Enlarged Cardiomediastinum
  - Pneumonia
  - Pneumothorax
- Pulmonary Parenchymal Abnormalities
  - Atelectasis
  - Consolidation
  - Lung Lesion
  - Lung Opacity
- Pleural Conditions
  - Pleural Effusion
  - Pleural Other
- Miscellaneous Findings
  - Fracture
  - Support Devices
  - No Finding

2 SUPPLEMENTARY RESULTS

2.1 Temporal Extension

In our main paper, we demonstrated the overall effectiveness of temporal simulation. Here, we further illustrate the effectiveness of `MediSim`'s temporal completion abilities by plotting the outpatient F1 Score against the visit number in Supplementary Figure 3. The plot shows that while the first few visits are less predictable, the accuracy quickly rises to a high level and remains consistently high. This indicates that during temporal extension, we only need a few visits to determine the type of record a patient has. Then we can effectively add additional visits to create a longer but still valid EHR.

2.2 Complete Downstream Enrichment Results

We provided downstream modeling results for `MediSim`-based simulated data on the most prevalent disease categories and an average over all categories in our main paper. For completeness, we provide a complete set of results for all categories and with all compared methods here. We provide the F1 Score for each model for each disease prediction task for our temporal extension experiment in Supplementary Table 3 and for our modality extension experiment in Supplementary Table 4. The columns are ordered by disease prevalence in ascending order. Note that for rare diseases with extreme label skew, we see most models (both unenriched and enriched) produce no positive predictions for the modality enrichment experiment, which drives the average results down. Overall, the results have large amounts of noise for low prevalence diseases there (`MediSim` outperforms unenriched data on 6 of the 8 highest prevalence tasks but far less on the rarer tasks). Still, they generally mirror those in the main paper. However, we can also see the general value of EHR enhancement through simulation. Most forms of temporal extension offer huge value over training

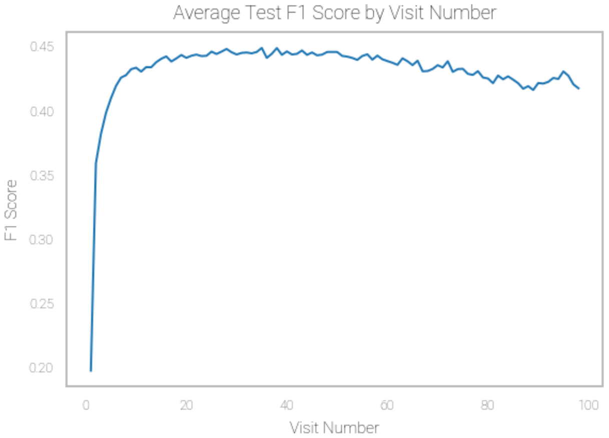

Supplementary Figure 3: Average next visit code prediction F1 Score by predicted visit number. The plot shows that while the first few visits are less predictable, the accuracy quickly rises to a high level and stays there permanently. This signifies that during temporal extension we only need a few visits to ascertain the type of record a patient has and then we can coherently and effectively add additional visits to form a longer but still viable EHR.

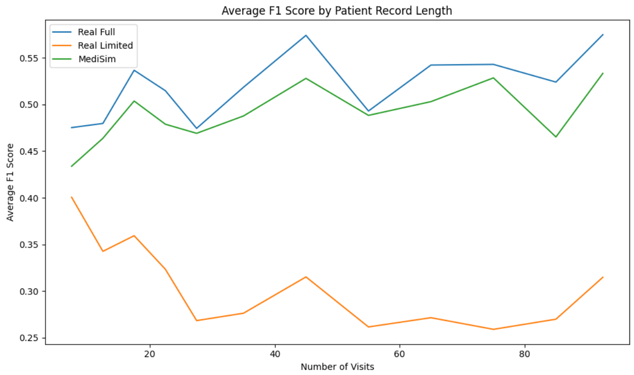

Supplementary Figure 4: Average F1 Score over our 10 different phenotype prediction tasks by the number of visits in the patient record. The performance by different training data demonstrates that while there is variability in effectiveness by record length, there is no significant bias in terms of this length induced by MediSim's temporal extension as it closely mirrors the pattern of performance of the real, full-length dataset.

on real but unenriched data with limited visits, as we commonly see in real-world settings. Specifically, all of the strong baselines (omitting LSTM, SynTEG, and RETAIN) provide a boost of at least 16% on average over the real, limited-length training data, and the GPT baseline even outperforms MediSim on average. We see a less consistent improvement in the modality enrichment baselines as MediSim outperforms the leading Neural Network baseline by 13% on average, more than over the unenriched data. However, even those baselines can help on some tasks, especially those with lower prevalence labels.

2.3 Temporal Enrichment Results by Record Length

While it is clear that MediSim and many other temporal extension baselines improve downstream performance on average by simulating additional patient visits, another concern beyond overall performance is the introduction of bias. Especially given the addition of extra visits, one worry is that this may reduce, or at least disproportionately improve, performance for patients with fewer visits. While we attempted to conceptually address this problem through Figure 2, showing that the distribution of the number of visits in MediSim's extended dataset closely matched that in the real, full-length dataset, we also provide more concrete analysis here. Specifically, we group the downstream test set by the number of visits (with buckets of size 5 starting at 5 visits through 30 visits before proceeding by steps of size 10 to the end) and show the average F1 score across our 10 tasks by the number of visits in the patient record under consideration. We show this across the models trained on the real, deficient dataset with limited-length records, the real, full-length dataset, and the MediSim-enriched dataset in Figure 4. There, we see that while the deficient data causes downstream models to struggle with more than the shortest records, the gold standard full-length real data and MediSim's enriched data allow for general consistency across record lengths. Furthermore, we see that MediSim does not induce any significant bias with respect to record length as it closely mirrors the variations in performance incurred by the models trained on real, full-length data (which are likely due to natural variance in difficulty due to the relatively small patient populations within each bucket).

2.4 Interchangeable Structure Modality Ordering

In our discussion section within our main paper, we referenced the interchangeability of different structured code modality orderings to argue for the validity of the diagnosis-procedure-medication ordering we conduct all our main experiments with, as well as to propose the possibility of having multiple simulation models to adapt to whatever format of missingness is encountered within a given dataset. To validate those arguments, we train and test a MediSim model on a reordered dataset that follows a medication-diagnosis-procedure ordering for each structured visit representation. We provide those results in Supplementary Table 5 where we compare them against our original results and see generally similar effectiveness. Specifically, the temporal performance is almost identical, while the decrease in modality extension performance is primarily a result of the difference in the modalities predicted (effectively a more difficult test set).

2.5 Downstream Task Hyperparameters

In our downstream enrichment evaluations, we also had two core hyperparameters that defined the problem settings of the temporal and modality enrichment experiments. We explore the effects of changing those hyperparameters here.

Supplementary Table 3: Complete Temporally Enriched Downstream Performance

	Alzheimer's	Osteoporosis	Stroke	Heart Failure	Arthritis	Chronic Kidney Disease	Heart Disease	COPD	Cancer	Diabetes	Average
Real, Limited-Length Data	0.255	0.084	0.227	0.406	0.103	0.343	0.360	0.319	0.388	0.674	0.316
GPT	0.479	**0.331**	**0.479**	0.541	0.287	**0.571**	**0.604**	**0.502**	**0.613**	0.776	**0.519**
LSTM	0.0	0.005	0.0	0.013	0.007	0.007	0.031	0.113	0.006	0.272	0.046
SynTEG	0.0	0.0	0.0	0.009	0.152	0.0	0.0	0.000	0.078	0.203	0.045
RETAIN	0.112	0.036	0.153	0.343	0.195	0.239	0.206	0.258	0.370	0.559	0.248
CONAN	0.333	0.166	0.360	0.450	0.268	0.584	0.555	0.519	0.509	0.776	0.453
Dipole	0.013	0.103	0.228	0.421	0.213	0.451	0.517	0.491	0.486	0.755	0.368
`MediSim`	**0.482**	0.263	0.418	**0.547**	**0.288**	0.540	0.585	0.486	0.585	**0.779**	0.498
Real, Full-Length Data	0.407	0.358	0.505	0.560	0.308	0.632	0.600	0.488	0.617	0.795	0.528

Supplementary Table 4: Complete Modality Enriched Downstream Performance

	Septicemia	Upper Respiratory Disease	Gastrointestinal Hemorrhage	Diabetes With Complications	Respiratory Failure	Renal Failure	COPD	Fluid Disorders	Essential Hypertension	Conduction Disorders	Other Liver Diseases	Hypertension	Lipid Disorders	Chronic Kidney Disease	Heart Disease	Cardiac Dysrhythmias	Heart Failure	Average
Real, Diagnosis-Only Data	0.0	0.0	0.0	0.195	0.069	0.0	0.074	0.0	0.406	0.210	0.355	0.442	0.210	0.482	0.416	0.588	0.666	0.242
Logistic Regression	0.0	**0.097**	0.0	0.0	0.0	0.0	0.0	0.0	**0.276**	0.195	0.355	0.222	**0.461**	0.434	0.613	**0.552**	0.681	0.239
Cascaded Residual Autoencoder	0.0	**0.148**	0.0	0.15	0.0	0.0	0.0	0.0	0.086	0.0	0.311	0.298	0.321	0.282	0.467	0.015	0.674	0.162
Neural Network	**0.023**	0.0	0.0	0.190	0.018	0.137	**0.126**	**0.028**	0.126	0.060	**0.448**	0.367	0.330	0.385	0.616	0.523	0.689	0.240
`MediSim`	0.0	0.074	0.0	**0.238**	**0.053**	**0.234**	0.037	0.0	0.015	**0.232**	**0.448**	**0.375**	0.358	**0.483**	**0.672**	0.524	**0.730**	**0.263**
Real, Full Modality Data	0.024	0.193	0.058	0.285	0.105	0.055	0.260	0.068	0.289	0.235	0.48	0.340	0.460	0.487	0.655	0.623	0.691	0.313

Supplementary Table 5: Inpatient Prediction Performance with Reordered Structured Modalities

	Temporal Extension		Modality Extension	
	Perplexity	F1 Score	Perplexity	F1 Score
Original `MediSim`	16.160	0.479	10.566	0.530
Reordered `MediSim`	16.197	0.459	36.019	0.356

For our temporal enrichment experiments, our limited-length data used 10 real visits, and we leveraged the temporal simulation models to simulate additional visits to enrich the data. This hyperparameter of the number of real visits can then be adjusted to provide more or less conditioning of simulated extension and to provide stronger or weaker limited-length data. So, we explore the effect of different real visit lengths on Average F1 Score test set performance in Supplementary Figure 5. There we see that while the patterns and orderings of models are generally the same, temporal simulation as a task gains greater value when fewer real visits are provided. This is due to the inability to train on limited-length data when we have less than 5 real visits (as the labels are generated based on the last 5 visits) and the steady improvement as more real visits are provided. Beyond that, the two strong methods (`MediSim` and GPT) are robust compared to other approaches as less real data is offered.

Similarly, for our modality enrichment experiments, we switched out a single real diagnosis code for each visit for another randomly selected code. This was meant to add noise to the diagnoses to force a greater dependence on the procedure and medication code modalities. However, this hyperparameter of several diagnosis codes swapped out can also be adjusted. So, we explore the effect of swapping either no diagnosis codes or additional codes on Average F1 Score test set performance in Supplementary Figure 6. There we see that without noise, the diagnosis-only data is as valuable as full-modality data and our `MediSim` enriched data. However, despite the

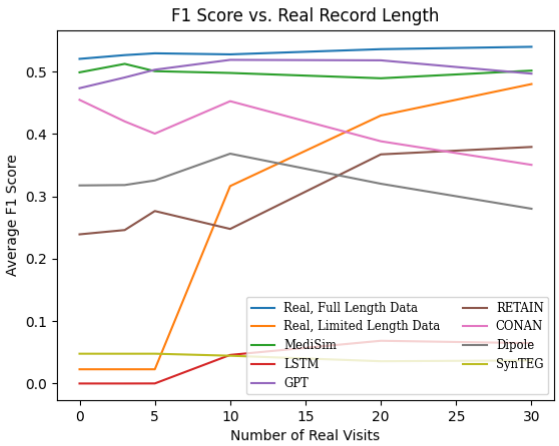

Supplementary Figure 5: Average F1 Score over our 10 different phenotype prediction tasks by the number of real visits to provide before extending the record via simulation. Temporal simulation as a task gains greater and greater value when fewer real visits are provided due to the inability of training on limited-length data when we have fewer than 5 real visits (as the labels are generated based on the last 5 visits) and the steady improvement of unenriched models as more real visits are provided. Beyond that, we see that `MediSim` and GPT are robust compared to other approaches as less real data is offered.

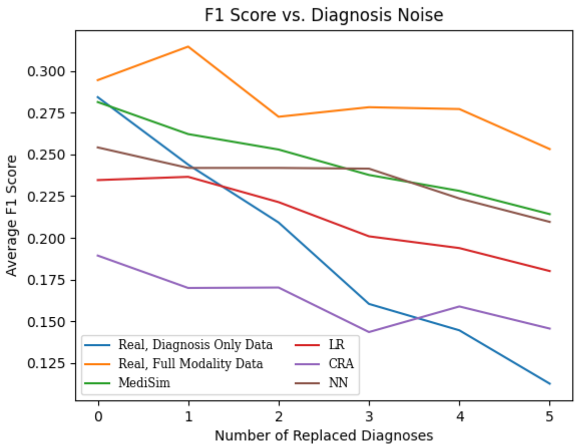

Supplementary Figure 6: Average F1 Score over our 17 different phenotype prediction tasks by the amount of diagnosis noise inserted into the patients. While adding noise quickly degrades the performance of models trained on diagnosis-only records, records enriched through modality imputation remain robust even to large amounts of noise. `MediSim` performs this simulation the best and outperforms other baselines at most noise levels.

variance within individual data points from the inherent randomness of the process, we see that the patterns from our main paper hold and even exaggerate as we add more noise. The diagnosis-only data continues to degrade in performance while `MediSim` rapidly and the neural network baseline remain robust via their simulated additional code modalities.

2.6 Additional Synthetic Note Examples

Our main paper displayed a single randomly selected pair of real-synthetic clinical note samples from our test set. However, we generated these samples for various temperature sampling levels for every visit in our test set. So, we display three more randomly selected pairs in our supplement in Supplementary Figure 7. There we see the same patterns as in our main text where the synthetic clinical notes can capture overall patterns of clinical note format and content and tune the generation to the specific content of the visit from the structured codes.

2.7 Additional Synthetic Image Samples

In our main paper we displayed a single randomly selected pair of real-synthetic medical image samples from our test set. However, we generated these samples for every image in our test set. So, we display four more randomly selected pairs in our supplement in Supplementary Figure 8. There, we see the same patterns as in our main text where the synthetic images can capture overall patterns of what Chest X-rays should look like and tune the generation to the specific structured information such as consolidation, pneumonia, lung lesions, and more.

Supplementary Table 6: Note-Enhanced Simulation Performance

	Temporal Extension		Modality Extension	
	Perplexity	F1 Score	Perplexity	F1 Score
`MediSim`	16.946	**0.434**	12.054	0.481
`MediSim` + Notes	**16.726**	**0.434**	**11.871**	**0.482**

2.8 Incorporating Complex Modalities

While we showed in our main paper the ability for `MediSim` to use X-ray imaging data to enhance its core structured code simulation, we replicate those results here for our multimodal clinical note dataset. Specifically, we train and compare a standard `MediSim` architecture ignoring the clinical note text, operating only on the structured data, against an enhanced `MediSim` architecture with note embedding models included. We present the results of those evaluations, looking exclusively at the visits after the first hospital stay (as they have clinical note embeddings to condition structured predictions on), in Supplementary Table 6. There, we see a slight albeit clear improvement in both temporal and modality prediction through the ability to leverage the information in clinical notes by encoding it to fit within the structured patient matrix representation.

We note that the more significant improvement in image-enhanced performance compared to note-enhanced performance is likely due to the greater value in the images above and beyond the past structured variables. The code variables in the MIMIC-CXR dataset are all imaging-based labels, and they are furthermore noisy based on

Real Clinical Note

admission date : [deidentified] discharge date : [deidentified] date of birth : [deidentified] sex : f service : cardiothoracic allergies : phenobarbital attending : [deidentified] chief complaint : s / p nstemi [deidentified] and had 3 vessel cad on cath. major surgical or invasive procedure : cabgx4 (lima - > lad, svg - > om, diag, plbr) [deidentified] history of present illness : 68 year old white female who presented to an outside hospital with doe and had an nstemi. she had a cath which showed 3 vessel cad and is now admitted for elective cabg. past medical history : htn s / p nstemi copd social history : sh : the patient smokes 2 ppd for the last 40 years. no etoh. works as a cashier at [deidentified] and lives alone. able to walk about 100feet before getting sob family history : fh : mi in mother in 40's physical exam : gen : elderly, white, female in nad. heent : nc / at, eomi, perla, oropharynx benign. neck : supple. from, no thyromegaly or lymphadenopathy, carotids 2 + = bil. without bruits. lungs : decreased bs bilat. cv : rrr without r / g / m abd : obese, soft, nontender, without masses or hepatosplenomegaly. ext : pulses 1 + = bilat throughout, 1 + bilat. pedal edema neuro : non - focal pertinent results : hematology complete blood count wbc rbc hgb hct mcv mch mchc rdw plt ct [deidentified] 06 : 40am 8. 3 3. 74 * 10. 2 * 30. 3 * 81 * 27. 4 33. 8 16. 7 * 193 basic coagulation (pt, ptt, plt, inr) plt ct [deidentified] 06 : 40am 193 chemistry renal & glucose glucose urean creat na k cl hco3 angap [deidentified] 06 : 40

Generated Clinical Note

Sample Text 1 11: admission date : [deidentified] discharge date : [deidentified] date of birth : [deidentified] sex : m service : cardiothoracic allergies : patient recorded as having no known allergies to drugs attending : [deidentified] chief complaint : s / p left arm major surgical or invasive procedure : [deidentified] cabg x 3 history of present illness : 52 yo male with h / o aortic insufficiency and hypertension presented to osh on [deidentified] with chest pain and ruled in for nstemi. troponin i of 1. 35. 0. past medical history : + tobacco use social history : current smokes 1ppd x 40 years etoh, denies other drug use family history : mother noted physical exam : wdwn m in nad, alert, overweight white male in bed skin unremarkable, well healed scar @cyte rash, no sternal incision c / d / i ; sternum stable, perrl, eomi, anicteric, rrr + ble edema 1 + bilat. her ble, bilat, k, 4 / 6 cva, 9 cm, i, d / i without erythema or drainage, + b / l, r. all le edema, nonfocal exam 2 + bilat. d, pt 2 + pulses. pertinent results : [deidentified] 06 : 35am blood wbc - 9. 9 rbc - 3. 38 * hgb - 9. 6 * hct - 25. 1 * mcv - 87 mch - 30. 1 mchc - 34. 5 rdw - 12. 6 plt ct - 246 [deidentified] 06 : 35am blood plt ct - 356 * [deidentified] 06 : 35am blood glucose - 100 urean - 8 creat - 1. 0 na - 139 k - 3. 9 cl - 100 hco3 - 25 angap - 9 [deidentified] 10 : 50am blood alt - 25 ast - 64 * ast - 18 alkphos - 32 * amylase - 81 * totbili - 1. 8 * radiology final report chest, ss, with no change in cath (t), sco3, and twi in the pre

Real Clinical Note

admission date : [deidentified] discharge date : [deidentified] date of birth : [deidentified] sex : m service : micu final diagnoses : 1. hyperglycemia. 2. hypophosphatemia. 3. hypokalemia. 4. myocardial ischemia. 5. autoimmune hemolytic anemia. 6. chronic lymphocytic leukemia. history of present illness : this is a 65 - year - old male with a history of polycythemia [deidentified], chronic lymphocytic leukemia, and autoimmune hemolytic anemia who presents with complaints of weakness. the patient state he has had a progressive fatigue which developed over the past three to four weeks. he also stated he has had dark urine for the past two weeks. he also reported subjective fevers times three weeks. review of systems : on review of systems, he denied headache but did report some night sweats. no arthralgias, myalgias, or abdominal pain. some nausea on the day of admission and some loose stools as well. no rashes. no dysuria. no cough, and some slight dyspnea on exertion. past medical history : 1. chronic lymphocytic leukemia. 2. polycythemia [deidentified]. 3. sarcoid. 4. autoimmune hemolytic anemia. 5. seizure disorder secondary to meningitis. 6. non - insulin - dependent diabetes mellitus. 7. depression. medications on admission : nph insulin, [deidentified], protonix, diflucan, paxil, reglan, dilantin, senna, trazodone. allergies : no known drug allergies. physical examination on presentation : in the emergency room, generally a male resting on stretcher, appeared tired but in no apparent distress. vital signs revealed temperature of 102. 2, pulse of 131, blood pressure of 142 / 63, oxygen saturation of 97 % on room air. head, eyes, ears, nose, and throat examination revealed normocephalic and atraumatic. pupils were equal and reactive to light and accommodation. extraocular muscles were intact. sclerae were anicteric. mucous membranes were moist. neck was supp

Generated Clinical Note

admission date : [deidentified] discharge date : [deidentified] date of birth : [deidentified] sex : m service : ccu history of present illness : the patient is a 50 year - old male with a past medical history significant for diabetes mellitus, coronary artery disease, status post myocardial infarction, status post recent hospitalization for acute myoclonic polyps, who was admitted on [deidentified], following his stay at that admission was complicated by many diabetes mellitus and an asystole and vision in the setting of a lipatrogenic polyc fasiculature, requiring interventional radiotherapy, and a myoclonoscopy showing polyps, and an esophagitis. the patient was discharged to rehabilitation on [deidentified] reconds in the emergency room with the circumstances in the ambulance and was admitted to the micu. on the day of admission the patient was found to be in acute renal failure with a potassium of 6. 1 and a bicarbonate level of 10. the patient was taken to the medical intensive care unit. past medical history : 1. diabetes mellitus type 2. chronic renal insufficiency. 2. hypertension. 3. hypercholesterolemia. 4. chronic renal insufficiency. 5. coronary artery disease status post three vessel cabg in [deidentified]. 6. noninsulin dependent diabetes mellitus. 7. carotid ultrasound in [deidentified]. 8. post cardiac catheterization in [deidentified]. 9. atrial fibrillation. 10. hypertension. 11. gout. 12. peripheral vascular disease. 13. left pelvic fracture, and ruptured labs. 14. anion gap of 8. allergies : penicillin. medications : 1. lithium. 2. digoxin 0. 25 units subcutaneously q. a. m. 3. regular insulin sliding scale. 4. diltiazem. 5. lipitor 10 mg p. o. b. i. d. 6. lopressor 25 mg p. o. b. i. d. 7. cellcept 1, 000 mg p. o. q. d. 8. ambien 5 mg p. o. q. d.

Real Clinical Note

admission date : [deidentified] discharge date : [deidentified] date of birth : [deidentified] sex : f service : surgery allergies : patient recorded as having no known allergies to drugs attending : [deidentified] chief complaint : possible bile leak and bleeding after lap chole major surgical or invasive procedure : [deidentified] exploratory laparoscopy with washout and drain replacement. history of present illness : 65y female s / p lap chole for acute cholecystitis pod # 5 c / b bile leak (pt had persistent pain and hida demonstrated leak) now is pod # 1 from return to or w / no evidence of active bile leak s / p ruq jp drain placement. she was transferred from [deidentified] hospital initially to ercp for potential stenting. however, jp drain had brb and brb soaked dressing. hct was 26. 8 but she was hemodynamically stable. jp hct 12. 7. ercp aborted and general surgery was initially consulted, patient was transferred to tsicu ; resuscitated with ivf x1l and prbcs x2. transplant surgery was then consulted given nature of potential injury. past medical history : dm ii (poorly controlled), htn, cva [deidentified] with residual left sided weakness, pud, h / o ugi bleed, chronic pancreatitis with calcifications, h / o anxiety / depression psh : laparoscopic cholecystectomy c / b bile leak [deidentified] social history : + tobacco, + etoh, denies recreational drugs. lives alone. independent in adl. family history : non - contributory physical exam : t 98. 5 p 99 bp 100 - 130 / 30 - 47 rr 19 o2sat 98 % on 3l nc gen : awake, alert, oriented only to self, nad heent : no scleral icterus cv : rrr pulm : ctab, no respiratory distress abd : decreased bs, soft, + distension, laparoscopic incisions w / staples c / d / i, no hematoma, no erythema, tenderness to palpation in ruq and along incisions, drain to

Generated Clinical Note

admission date : [deidentified] discharge date : [deidentified] date of birth : [deidentified] sex : f service : surgery allergies : amoxicillin / codeine / peanuts attending : [deidentified] chief complaint : abdominal pain major surgical or invasive procedure : [deidentified] procedure : 1. percutaneous tracheostomy [deidentified] percutaneous tracheostomy with attempted central venous catheter placement [deidentified] 1. ptc placement [deidentified] 1. placement of percutaneous tissue and p external state of right palate lobe. 2. omental tissue and 8 - thickness gj tube placement [deidentified] 1. tracheostomy and percutaneous tracheostomy tube placement history of present illness : 35f who presented to the [deidentified] ed on [deidentified] with abdominal pain, hematoma, and jaundice. at the osh, a symmetrical colic b / lmisliteration transpositioning anemia with abd distension, and a 1. 5cm. her labs were notable for wbc = 11. 9 with 22 % bandemia, hct 29. 8, lfts were alt / ast was 5th, alt 142, tbili 9. 6, alk, inr 10. 9, tbili 2. 5. 4. abdominal ct was performed to be a large distended cc of fluid that was negative for acute pancreatitis into the duodenum. a retroperitoneal mass was noted to be a pancreatic involvement. she was again admitted to an osh on [deidentified] for ards. a repeat ct scan demonstrated a large pancreatic mass with mass effect. two stomach collapse were also noted at the level of the pancreas distal pancreatic head (likely). she was then transferred to [deidentified] for further management.. in the ed, initial vs were : 98. 6, 66, exams, were consistent with arf but had arf resolved. on the [deidentified] she was given a total of 12 units of ivf and started on ivf and levophed (at osh) and flagyl was added.. cxr did not show any acute infifi

Supplementary Figure 7: Three more randomly selected pairs of real (left) and synthetic (right) clinical notes each based on the same structured medical code information

the content covered within the clinician's summary report. So, including past imaging embeddings can provide valuable and highly relevant information not present in the structured history. However, the discharge summary text is often unable to cover all of a patient's structured data, applying only to a subset of the greater than 10,000 medical code variables we are simulating. So, greater applicability of the complex modality and greater unreliability of the structured variables increase the performance gain from leveraging these complex modalities within our `MediSim` framework.

SUPPLEMENTARY REFERENCES

[1] Johnson, A. *et al.* Mimic-cxr-jpg-chest radiographs with structured labels. *PhysioNet* (2019).

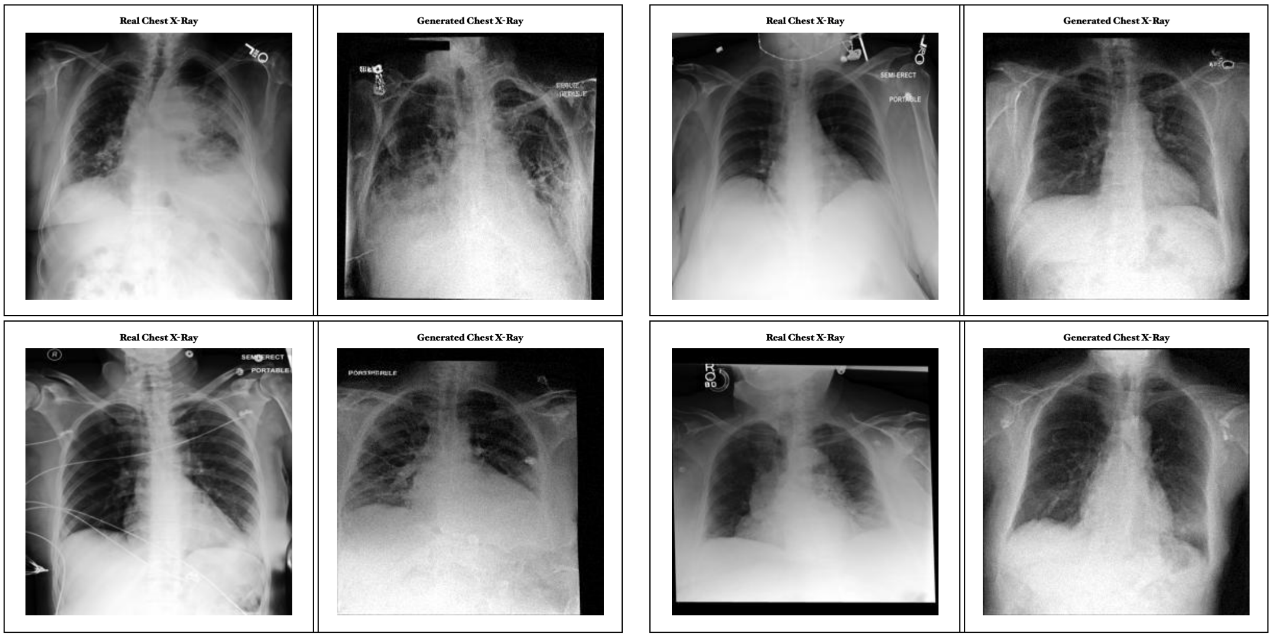

Supplementary Figure 8: Four more randomly selected pairs of real and synthetic medical images each based on the same structured medical information
